# Development and validation of a rapid five-minute nucleic acid extraction method for respiratory viruses

**DOI:** 10.1186/s12985-024-02381-3

**Published:** 2024-08-18

**Authors:** Yu Wang, Yuanyuan Huang, Yuqing Peng, Qinglin Cao, Wenkuan Liu, Zhichao Zhou, Guangxin Xu, Lei Li, Rong Zhou

**Affiliations:** 1 Guangzhou National Laboratory, Guangzhou, China; 2 State Key Laboratory of Respiratory Disease, National Clinical Research Center for Respiratory Disease, Guangzhou Institute of Respiratory Health, the First Affiliated Hospital of Guangzhou Medical University, Guangzhou Medical University, Guangzhou, China; 3 School of Pharmacy, Tongji Medical College, Huazhong University of Science of Technology, Wuhan, China; 4 GIRM Biosafety (Guangzhou) Co., Ltd, Guangzhou, China

**Keywords:** Respiratory viruses, Glycerin and ethanol, Five-minute nucleic acid extraction

## Abstract

**Background:**

The rapid transmission and high pathogenicity of respiratory viruses significantly impact the health of both children and adults. Extracting and detecting their nucleic acid is crucial for disease prevention and treatment strategies. However, current extraction methods are laborious and time-consuming and show significant variations in nucleic acid content and purity among different kits, affecting detection sensitivity and efficiency. Our aim is to develop a novel method that reduces extraction time, simplifies operational steps, and ensures high-quality acquisition of respiratory viral nucleic acid.

**Methods:**

We extracted respiratory syncytial virus (RSV) nucleic acid using reagents with different components and analyzed cycle threshold (Ct) values via quantitative real-time polymerase chain reaction (qRT-PCR) to optimize and validate the novel lysis and washing solution. The performance of this method was compared against magnetic bead, spin column, and precipitation methods for extracting nucleic acid from various respiratory viruses. The clinical utility of this method was confirmed by comparing it to the standard magnetic bead method for extracting clinical specimens of influenza A virus (IAV).

**Results:**

The solution, composed of equal parts glycerin and ethanol (50% each), offers an innovative washing approach that achieved comparable efficacy to conventional methods in a single abbreviated cycle. When combined with our A Plus lysis solution, our novel five-minute nucleic acid extraction (FME) method for respiratory viruses yielded superior RNA concentrations and purity compared to traditional methods. FME, when used with a universal automatic nucleic acid extractor, demonstrated similar efficiency as various conventional methods in analyzing diverse concentrations of respiratory viruses. In detecting respiratory specimens from 525 patients suspected of IAV infection, the FME method showed an equivalent detection rate to the standard magnetic bead method, with a total coincidence rate of 95.43% and a kappa statistic of 0.901 (*P* < 0.001).

**Conclusions:**

The FME developed in this study enables the rapid and efficient extraction of nucleic acid from respiratory samples, laying a crucial foundation for the implementation of expedited molecular diagnosis.

**Supplementary Information:**

The online version contains supplementary material available at 10.1186/s12985-024-02381-3.

## Introduction

Acute respiratory virus infection is a prevalent human disease, particularly affecting children, the elderly, and immunocompromised individuals, with a high incidence during certain seasons [1, 2]. Common respiratory viruses include respiratory syncytial virus (RSV), adenovirus (ADV), influenza virus, and severe acute respiratory syndrome coronavirus 2 (SARS-CoV-2) [3]. Among them, influenza A virus (IAV) and SARS-CoV-2 have caused global pandemics, posing a serious threat to human health and social public safety [4, 5]. Currently, ongoing research and development for RSV and SARS-CoV-2 vaccines are being conducted worldwide; however, achieving herd immunity will still be a prolonged process due to challenges such as low vaccination rates and virus mutations in low- and middle-income countries [6, 7].

The nucleic acid amplification test (NAAT) and its detection of specific DNA is a powerful tool widely employed in various fields, including disease diagnosis, gene functional analysis, and mutation screening. In diagnostic applications, NAAT-based analysis offers several advantages over traditional enzyme or antibody-based methods, including higher sensitivity, faster sample-to-result processing time, and greater flexibility in target detection selection, which enables rapid adaptation to various emerging challenges [8]. However, the major bottleneck preventing the rapid and large-scale implementation of molecular diagnostics is the separation and purification of nucleic acid from samples, which is a complex process that traditionally requires skilled technicians and involves numerous manual pipetting steps [9].

Isolating high-quality nucleic acid while preserving its purity and integrity is an essential prerequisite for downstream molecular applications, including quantitative real-time polymerase chain reaction (qRT-PCR), isothermal amplification technologies (IATs), next-generation sequencing (NGS), and microarrays [10–13]. Although there are several methods for nucleic acid isolation, they can be primarily categorized into three groups: liquid-phase extraction utilizing phenol and guanidine thiocyanate (GTC), solid-phase adsorption employing silica membrane spin columns, and superparamagnetic beads. The methods all follow a common sequence: first, cell lysis and inhibition of ribonuclease P (RNase P) activity with subsequent separation of nucleic acids from the lysed mixture; then, washing of the nucleic acids; and, ultimately, recovery of purified nucleic acids [14]. After phenol-based TRIzol reagent is used to extract nucleic acids, they undergo separation and purification through precipitation. This method is widely used for RNA extraction from tissues and cells; however, TRIzol requires a duration exceeding 70 min for extraction, the recovered RNA often suffers from contamination due to residual organic matter [15]. The spin column method is based on the binding of nucleic acids to a solid-phase silica carrier under a high salt concentration, followed by a series of washing and centrifugation steps to eliminate contaminants, and finally elution of the nucleic acids from the silica using a low-salt solution [16]. The spin column method involves numerous operational steps, with a duration of approximately 40–60 min, and requires multiple centrifugations during extraction, which increases the risk of nucleic acids breakage and degradation. The magnetic bead method employs paramagnetic beads coated with various functionalized surface chemicals to efficiently capture and purify nucleic acids. In addition, the magnetic beads are separated from the liquid during washing and elution steps by utilizing a magnet to attract the beads [17, 18]. The magnetic bead method only requires 25–30 min, but the recovery rate of nucleic acid in the eluent is relatively low, and the presence of residual magnetic beads also exerts an inhibitory effect on subsequent PCR [19]. The extraction process can be broadly divided into manual and automatic processes, with many DNA/RNA extraction kits designed for manual operation. However, with the increasing demand for high-throughput analysis, there is a growing popularity of kits specifically designed for automatic operation.

Given the instability of RNA and the ubiquitous presence of RNA enzymes, it is crucial to select an RNA assay kit that can simultaneously meet quality, purity, and integrity requirements while minimizing the time required. In this study, we have developed a rapid respiratory virus nucleic acid extraction method that can be completed within 5 min. The method not only ensures the quality of the extracted nucleic acid but also reduces processing time, thereby facilitating prompt and efficient downstream applications based on nucleic acid analysis.

## Materials and methods

### Cells, viruses, and clinical specimens

AD293 cells (from American Type Culture Collection) were cultured in Dulbecco’s modified Eagle medium (DMEM; Gibco, Grand Island, NY, USA) supplemented with 10% fetal bovine serum (FBS; Gibco, Grand Island, NY, USA), 1% penicillin, and 1% streptomycin (Gibco, Grand Island, NY, USA) at 37°C in an incubator with a 5% CO_2_ atmosphere. RSV (RSV-A2), ADV (ADV4), IAV (H1N1), herpes simplex virus 1 (HSV-1), and human coronavirus 229E (HCoV-229E) were obtained from the State Key Laboratory of Respiratory Disease. A pseudovirus of SARS-CoV-2 was purchased from Sansure Biotech Inc (Changsha, China). Frozen clinical specimens from 525 suspected IAV-infected patients were obtained from the First Affiliated Hospital of Guangzhou Medical University. This project was approved by the biosafety committee, and all experiments were performed in accordance with biosafety regulations.

### DNA and RNA extraction

We have developed a five-minute nucleic acid extraction (FME) reagent (China Food and Drug Administration (CFDA) Certification Class I, No. 20230661). The reagents comprised a lysis solution containing GTC, sodium citrate tribasic dihydrate (sodium citrate), sodium lauroyl sarcosine (sarkosyl), dithiothreitol (DTT), polyethylene glycol 6000 (PEG 6000), and isopropyl alcohol (IPA); a washing solution consisting of a mixture of glycerin and ethanol (EtOH) in equal proportions; an elution solution composed of Tris–HCl (pH 8.0) and ethylene diamine tetraacetic acid (EDTA); and magnetic beads purchased from BayBio Bio-tech Co., Ltd (Guangzhou, China). A total of 40 μL of magnetic beads and 500 μL of lysis solution were added to a 1.5 mL centrifuge tube, followed by the addition of 200-μL samples that were mixed by vortexing for 1 min, and subsequently all the supernatant was removed using a magnetic separator. The magnetic beads were washed with 300 μL of washing solution, vortexed for 1 min, and subjected to magnetic separation again. The supernatant was discarded. Then, 100 μL of elution solution was added, the mixture was incubated at 56°C for 1 min and magnetically separated, and all the supernatant containing the extracted nucleic acid was transferred. Alternatively, an automated nucleic acid extractor can be used by adding a 200-μL sample to a preloaded well plate containing reagents and placing it into an E-Five nucleic acid extractor (HuYanSuo Medical Technology Co., Ltd., Guangzhou, China). The machine was operated according to the manufacturer’s instructions, with the entire process taking approximately 5 min. Upon completion of the extraction, 100 μL of eluent was stored at −80°C for further use.

To compare the efficiency of extraction, the same sample was extracted according to the manufacturer’s instructions for each commercial reagent kit while maintaining consistent loading and elution volumes. The extraction kits utilized in this study included the following: LemnisCare (LC) Viral DNA/RNA Extraction kit (LemnisCare Medical Technology Co., Ltd., Shenzhen, China), referred to as LC; Magen Total DNA/RNA kit (Magen Biotechnology Co., Ltd., Guangzhou, China), referred to as Magen; Baypure Viral DNA/RNA Extraction kit (BayBio Bio-tech Co., Ltd., Guangzhou, China), referred to as Baypure; HR Fast-Virus DNA/RNA kit (Huirui Biotechnology Co., Ltd., Zhuhai, China), referred to as HR; Viral Nucleic Acid Isolation kit (Jiangsu BioPerfectus Technologies Co., Ltd., Taizhou, China), referred to as BioPerfectus; TIANamp Virus DNA/RNA kit (Tiangen Biotech Co., Ltd., Beijing, China), referred to as TIANamp; ABT Nucleic Acid Extraction kit (Applied Biological Technologies Co., Ltd., Beijing, China), referred to as ABT; GIRM Nucleic Acid (DNA/RNA) Extraction kit (HuYanSuo Medical Technology Co., Ltd., Guangzhou, China), referred to as GIRM; and TRIzol reagent (Thermo Fisher Scientific, Waltham, MA, USA), referred to as TRIzol.

### Nucleic acid concentration and integrity

The concentration and purity of RNA from each kit were assessed by analyzing 1 μL of extracted nucleic acid using the NanoDrop One (Thermo Fisher Scientific, Waltham, MA, USA). A known concentration of plasmids was added to the FME to compare the amount of DNA recovered from the elution solutions while concurrently assessing the integrity of the DNA fragments through 1.5% agarose gel electrophoresis.

### Quantitative real-time PCR

The qRT-PCR experiment was conducted using a SLAN-96P real-time PCR detection system (Hongshi Medical Technology Co., Ltd., Shanghai, China). The RSV, ADV, IAV, and pseudovirus of SARS-CoV-2 were analyzed with a respiratory syncytial virus Nucleic Acid Diagnostic kit (HuYanSuo Medical Technology Co., Ltd., Guangzhou, China), human adenovirus Nucleic Acid Diagnostic kit (HuYanSuo Medical Technology Co., Ltd., Guangzhou, China), SARS-CoV-2 and influenza A/B Virus Nucleic Acid Diagnostic kit (Sansure Biotech Inc., Changsha, China), and 2019-nCoV Nucleic Acid Diagnostic kit (Sansure Biotech Inc., Changsha, China), respectively. The reaction system and procedures of the four commercially available test kits were followed according to the instructions. HSV-1 and HCoV-229E were detected using SYBR Green dye (Tsingke Biotech Co., Ltd., Beijing, China). The nucleic acid of HCoV-229E required an additional reverse transcription step of 25°C for 5 min, activation at 50°C for 15 min, and 85°C for 2 min (Vazyme Biotech Co., Ltd., Nanjing, China). The reaction system consisted of 10 μL of ArtiCan^ATM^SYBR qPCR Mix (Tsingke Biotech Co., Ltd., Beijing, China), 1.5 μL of forward and reverse primers (Additional file 1: Table S1), and 7 μL of DNA template. The PCR cycling program was as follows: 95°C for 1 min; and 40 cycles of 95°C for 10 s, 60°C for 20 s, followed by melting curve analysis.

### Clinical performance of the FME

Frozen respiratory tract specimens (pharyngeal swabs) were collected from suspected IAV-infected patients, and the FME and standard magnetic bead method (BioPerfectus) were used for nucleic acid extraction. Both extraction methods ensured consistent sample volumes of 200 μL and elution volumes of 100 μL. The SARS-CoV-2 and Influenza A/B Virus Nucleic Acid Diagnostic kits (Sansure Biotech Inc., Changsha, China) were used for qRT-PCR detection, and specifically for the detection of IAV clinical specimens. Briefly, 20 μL of nucleic acid was added to a mixture containing 26 μL of a PCR buffer mixture (dNTPs, MgCl_2_, primer, and probe) and 4 μL of an enzyme mixture (reverse transcriptase and Taq polymerase). The PCR cycling program was as follows: 50°C for 4 min and 95°C for 30 s, followed by 45 cycles of 95°C for 2 s and 60°C for 20 s. The SLAN-96P system (Hongshi Medical Technology Co., Ltd., Shanghai, China) was used for qRT-PCR. A VIC channel cycle threshold (Ct) value of ≤ 40 and a simultaneous Cy5 channel Ct value of ≤ 40 indicated positivity for IAV. If at least one of the two extraction methods yielded a positive result for IAV, the sample was classified as positive for IAV; otherwise, it was considered negative.

### Quantitative analysis of proteins

Modified Bradford reagent (Sangon Biotech Co., Ltd., Shanghai, China) was used to quantify the protein content in the washing solution after a magnetic bead wash. In brief, the reagent was used to measure the OD595 of bovine serum albumin (BSA) with a range of known concentrations. A standard curve correlating the absorbance with protein concentration at 595 nm was constructed. Subsequently, the OD595 of the test sample was measured in the same manner to determine its protein concentration by referencing the standard curve.

### Statistical analysis

SPSS Statistics 20 software (IBM, Armonk, NY, USA) was used to calculate the percentage of positive and negative agreements between the FME and standard magnetic bead methods, and Cohen’s kappa statistic was calculated [20]. Statistical graphs were generated using GraphPad Prism 8 (GraphPad Software Inc., San Diego, CA, USA). Statistical analysis was performed with Student’s *t* tests, and *P* < 0.05 (two tailed) was considered statistically significant. The data presented are representative of at least three independent experiments.

## Results

### The combination of glycerin and EtOH can efficiently purify nucleic acid

**Fig. 1:**
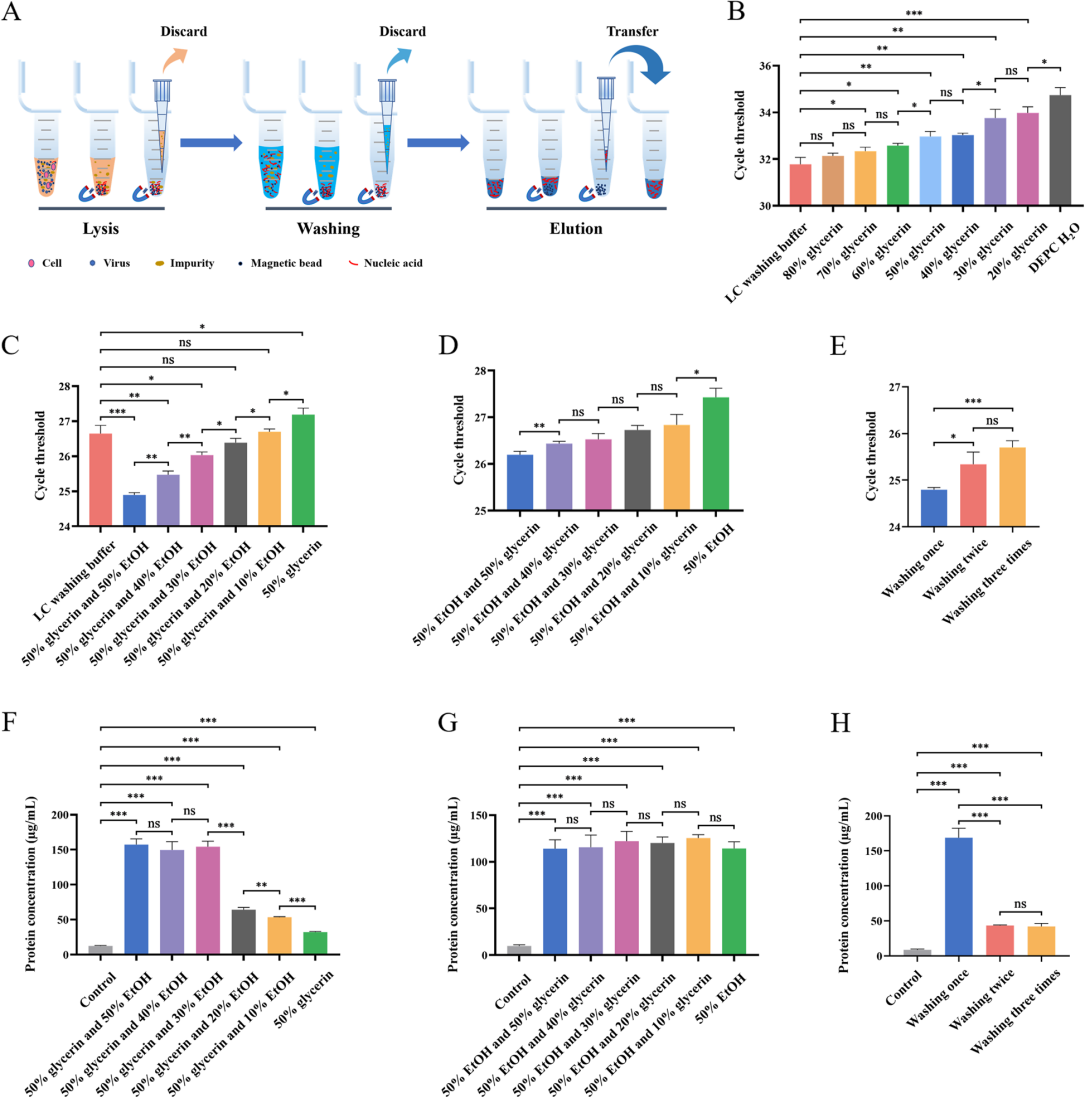
Glycerin and EtOH combination for nucleic acid purification. **A** Schematic diagram of the nucleic acid extraction magnetic bead method. **B** Using the LC reagent as a control group, varying glycerin concentrations (80%, 70%, 60%, 50%, 40%, 30%, and 20%) and diethyl pyrocarbonate (DEPC) H_2_O were used to replace the LC kit washing buffer for low-concentration RSV nucleic acid extraction. One washing was performed, and qRT-PCR Ct values were used to assess the washing solution effect. **C** Using the LC reagent as a control group, different glycerin–EtOH combinations (50% glycerin and 50% EtOH, 50% glycerin and 40% EtOH, 50% glycerin and 30% EtOH, 50% glycerin and 20% EtOH, 50% glycerin and 10% EtOH, and 50% glycerin) were used to replace the LC kit washing buffer for high-concentration RSV nucleic acid extraction. One washing was performed, and qRT-PCR Ct values were used to assess the washing solution effect. **D** EtOH-glycerin solutions (50% EtOH and 50% glycerin, 50% EtOH and 40% glycerin, 50% EtOH and 30% glycerin, 50% EtOH and 20% glycerin, 50% EtOH and 10% glycerin, and 50% EtOH) were used to replace the LC kit washing buffer for high-concentration RSV nucleic acid extraction. One washing was performed, and qRT-PCR Ct values were used to assess the washing solution effect. **E** High-concentration RSV nucleic acid extraction using 50% glycerin and 50% EtOH washing solution, followed by one, two, or three rounds of purification in the washing step. Efficiency was evaluated based on qRT-PCR Ct values. **F**–**H** Wash solutions obtained from the wash steps in C–E were analyzed for the protein concentration. Data are presented as means ± SD for three independent biological replicates. Statistical significance was calculated using *t* tests; ns *P* > 0.05, *P < 0.05, **P < 0.01, ***P < 0.001

Nucleic acid extraction by the magnetic bead method involves three steps: lysis, washing, and elution (Fig. 1A). To develop a rapid and efficient method for nucleic acid extraction, we have explored a novel composition of lysate and washing solution with magnetic beads. First, the LC kit was selected as the control group due to its efficiency being comparable to that of other commercial kits for RSV and ADV extraction (Additional file 2: Figure S1), and a novel washing solution, glycerin, was substituted for the one in the LC kit to compare its washing efficacy to that of this LC washing buffer. Glycerin, a commonly employed protein stabilizer and storage buffer component, was identified as a viable washing solution for the purification of nucleic acid on magnetic beads in our study. As the glycerin concentration increased gradually from 20% to 80%, the extraction efficiency of RSV gradually improved, with no significant difference between 80% glycerin and the LC washing buffer (Fig. 1B). Considering the high viscosity of concentrated glycerin, we chose 50% glycerin and gradually increased the EtOH concentration on this basis. The results showed that compared with 50% glycerin, the higher the EtOH content, the better the extraction efficiency of RSV. When the proportion of EtOH reached 30%, its washing effect surpassed that of the LC washing buffer, reaching optimal performance when the EtOH concentration increased to 50% (Fig. 1C). Subsequently, we gradually increased the glycerin content while maintaining 50% EtOH and observed a corresponding gradual enhancement in extraction efficiency as the glycerin increased from 10% to 50% (Fig. 1D). This showed that the combination of 50% glycerin and 50% EtOH allowed glycerin and EtOH to effectively perform their respective washing functions and that the solution with this ratio possessed an optimal viscosity for facilitating the subsequent steps. Furthermore, it was surprising to observe that using 50% glycerin and 50% EtOH for one wash yielded better results than washing two or three times (Fig. 1E). This indicated that a single wash with 50% glycerin and 50% EtOH effectively eliminated the majority of impurities, while increasing the number of washes may result in the loss of nucleic acids. To further explore the efficacy of glycerin in removing impurities, we quantitatively measured the protein content of the novel washing solution after the washing process. The presence of 50% glycerin was found to exhibit a certain protein elution effect; however, as the proportion of EtOH gradually increased and reached 30%, the efficiency of the protein elution was significantly enhanced (Fig. 1F). The proportion of glycerin was gradually increased while maintaining 50% EtOH, but it was found that the washed protein content remained relatively stable (Fig. 1G), suggesting that while glycerin removed impurities, the primary ability to wash proteins was due to EtOH. Simultaneously, we also found that washing only once with 50% glycerin and 50% EtOH effectively eliminated the majority of protein impurities, while subsequent rounds of washing yielded less protein removal.

### The effect of A Plus lysis solution

**Fig. 2:**
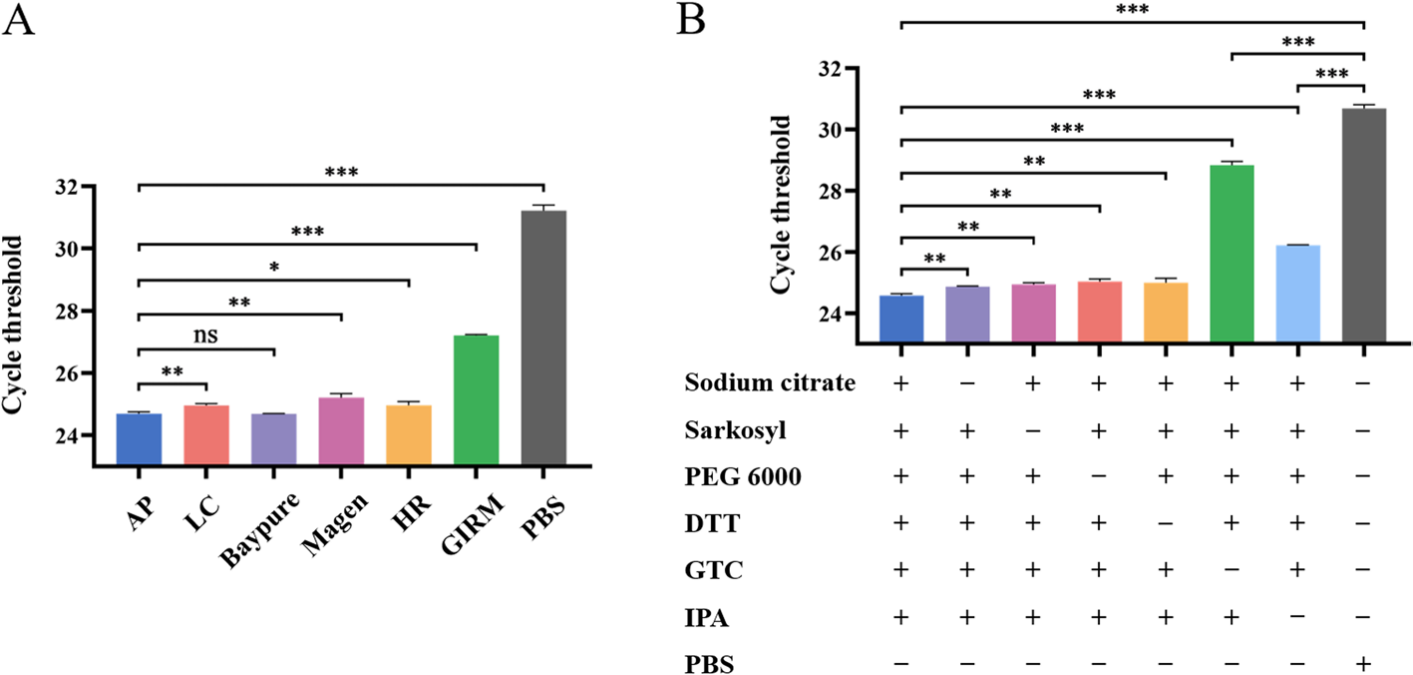
The AP solution demonstrates high efficacy in sample lysis. **A** During the extraction of high-concentration RSV nucleic acid, the lysis solution was prepared using either AP, phosphate-buffered saline (PBS), or a commercially available nucleic acid extraction kit lysis buffer. The washing solution consisted of a mixture of 50% glycerin and 50% EtOH, and the efficiency of each lysis solution was determined by analyzing the qRT-PCR Ct values. **B** AP lysis solution was prepared lacking specific components, and these lysis solutions or PBS were used to extract RSV viral nucleic acid at high concentrations. Subsequently, a wash step was performed using 50% glycerin and 50% EtOH, followed by the determination of qRT-PCR Ct values to assess the efficiency of each lysis solution. Data are presented as means ± SD for three independent biological replicates. Statistical significance was calculated using *t* tests; ns *P* > 0.05, *P < 0.05, **P < 0.01, ***P < 0.001

In addition to the washing solution, we also developed a novel lysis solution termed A Plus (AP). When using AP or other commercial lysis buffers combined with 50% glycerin and 50% EtOH to extract high-concentration RSV, the lysis effect of AP was essentially comparable or even slightly superior to that of other brands of lysis buffer (Fig. 2A), indicating the superior effectiveness of AP compared to the lysate in other commercially available kits. To investigate the key components involved in AP lysis, we used AP that lacked individual components to re-extract RSV. The results indicate that, compared with the intact AP, the reduction of each component in the AP lysis solution led to a decrease in extraction efficiency, among which the most significant impact was caused by reducing GTC or IPA (Fig. 2B), indicating that the reduction of any constituent of the AP lysis solution adversely affects the final extraction effect, with the absence of GTC and IPA having the most pronounced impact. This suggested that GTC and IPA play a pivotal role in the lysis effect of AP solution. If a lysis solution is not thoroughly washed during the extraction process, it may have a significant impact on subsequent experiments, such as PCR amplification. Therefore, we also investigated whether adding various components of AP to the elution solution affected qRT-PCR performance. We found that the amplification of qRT-PCR was completely inhibited after the direct addition of AP lysis solution or GTC, and sarkosyl also had a significant inhibitory effect (*P* < 0.01). Although the degree of inhibition was not obvious with sodium citrate, it decreased the fluorescence normalized reporter (Rn) value of qRT-PCR (Additional file 3: Figure S2A and S2B). Further research showed that the inhibitory effect of sodium citrate, sarkosyl, and GTC on qRT-PCR disappeared when their concentration in the elution solution was reduced to 1.25, 1, and 0.1 mM, respectively (Additional file 3: Figure S2C–S2H). This implied that while sodium citrate, sarkosyl, and GTC are the primary components in the lysis process, they must be removed during the washing stage to ensure that their concentration in the elution solution remains below inhibitory levels.

### Extraction effect of the FME

**Fig. 3:**
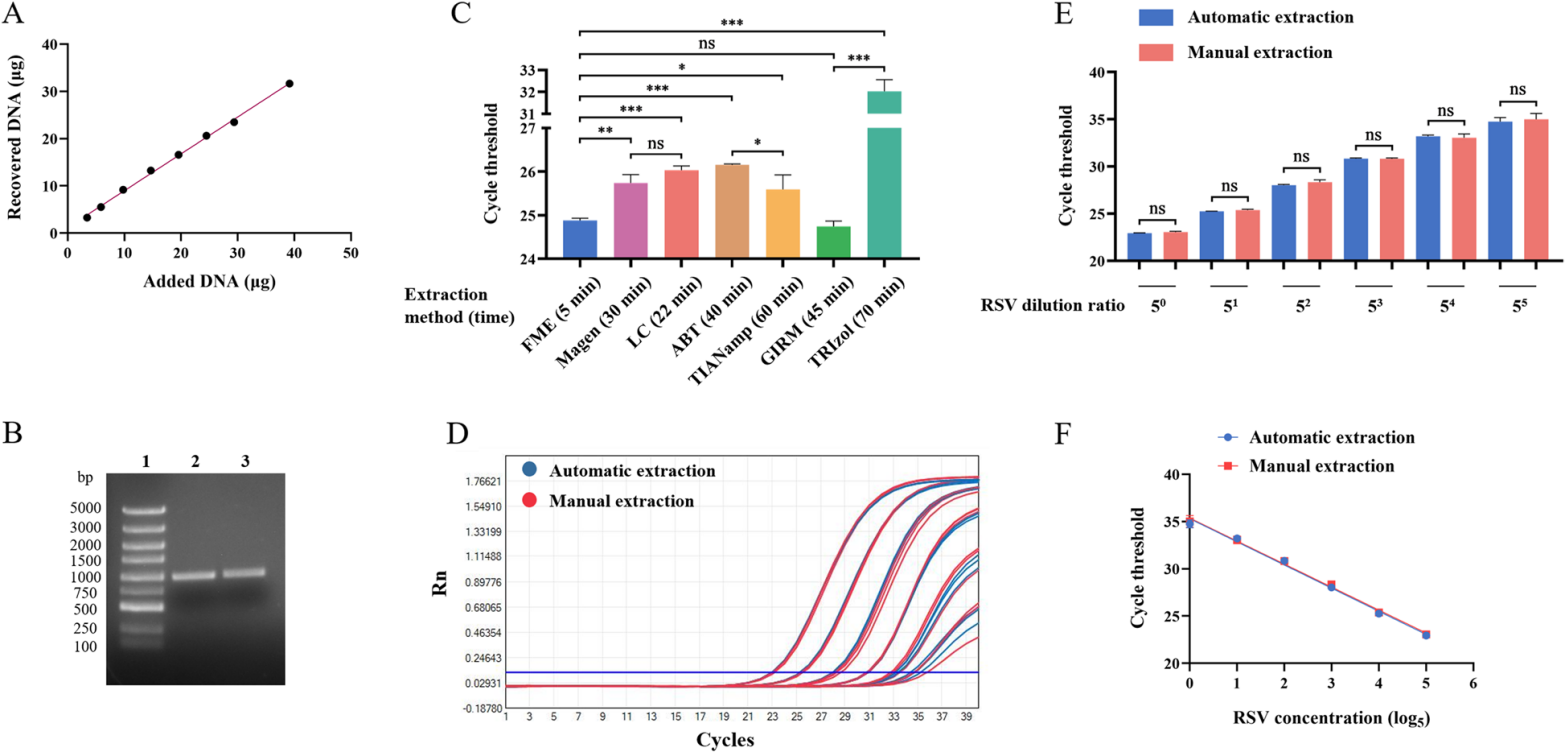
Performance analysis of the FME method. **A** There was a strong linear correlation (Pearson’s *R*, *R*^*2*^ = 0.9969) between the amount of DNA added to the FME and the amount of DNA recovered from the FME (μg). **B** DNA integrity was analyzed by agarose gel electrophoresis. Line 1: DNA Marker, Line 2: DNA before being added to the FME, Line 3: DNA after being added to the FME. **C** The FME and six different nucleic acid extraction kits were used to extract high-concentration RSV, and the Ct values obtained through qRT-PCR served as the criteria for evaluating the efficiency of each extraction kit. **D–F** PBS was used for fivefold gradient dilutions of RSV, and each gradient was subjected to nucleic acid extraction using either manual or automatic methods. The Ct values were used to assess the consistency between automatic and manual operations. D: Amplification curve, E: histogram, F: linear regression curve, manually extracted *R*^*2*^ = 0.9921, and automatically extracted *R*^*2*^ = 0.9922. Data are presented as means ± SD for three independent biological replicates. Statistical significance was calculated using *t* tests; ns *P* > 0.05, *P < 0.05, **P < 0.01, ***P < 0.001

We used AP lysis solution, washing solution (50% glycerin and 50% EtOH), magnetic beads, and an elution solution separately and sequentially to form the FME. The concentration and purity of RNA are two important criteria for any RNA extraction process. We found that the concentration of RNA obtained after applying the FME method to AD293 cells was higher than that of other traditional methods, and the purity (260/280 ratio) remained between 1.9 and 2.0 (Table 1). Recovery and integrity are metrics used to evaluate the effectiveness of nucleic acid extraction systems. To evaluate the extraction efficiency of the FME on DNA, we added known amounts of DNA to AP and compared it with the amount of DNA in the elution after extraction. The results showed that there was a strong linear correlation between the amounts of DNA added to the FME and the amounts of DNA recovered from the system (Fig. 3A) and that complete nucleic acid fragments were obtained through extraction using the FME (Fig. 3B). Subsequently, we also conducted a comparative analysis of the RSV extraction efficiency using the FME and several commercially available kits, including magnetic bead (Magen and LC), spin column (ABT and TIANamp), and precipitation methods (GIRM and TRIzol). The results showed that both the FME and GIRM precipitation methods yielded the highest RSV nucleic acid concentrations, with FME demonstrating a shorter processing time, and the FME was superior to the magnetic bead method and spin column method reagents used as a comparison (Fig. 3C). This suggested that the FME could obtain a high RNA concentration from viral samples in a short period of time.

**Table 1:** The concentration and purity of RNA extracted with different nucleic acid extraction kits

Method	RNA concentration (mean±SD) (ng/μL)	RNA purity (mean±SD) (260 nm/280 nm)
FME	33.76±0.52	1.92±0.01
Magen	19.81±0.14	1.78±0.01
LC	10.15±0.09	1.97±0.05
ABT	26.21±0.31	1.95±0.01
TIANamp	16.19±0.78	1.93±0.03
GIRM	11.28±0.54	1.97±0.03
TRIzol	32.35±2.23	1.49±0.00

### Automation and performance evaluation of the FME

Automation and high throughput are two of the biggest advantages of the magnetic bead method [21]. Considering the differences in RNA yield between manual and automatic extraction, we paired the FME with an automatic nucleic acid extraction instrument and implemented specific procedures to achieve a 5-minute extraction (Additional file 1: Table S2) and then compared the differences between the manual and automatic FME. As a result, it was observed that there was no significant difference in the efficacy of the manual or automatic FME when using fivefold diluted RSV samples sequentially (Fig. 3D and E), and the regression curves obtained from both approaches exhibited a high degree of overlap (Fig. 3F). This showed that after using a specific program, the FME could be adapted to currently available instruments and consumables, achieving high-quality automatic nucleic acid extraction within 5 min.

**Fig. 4:**
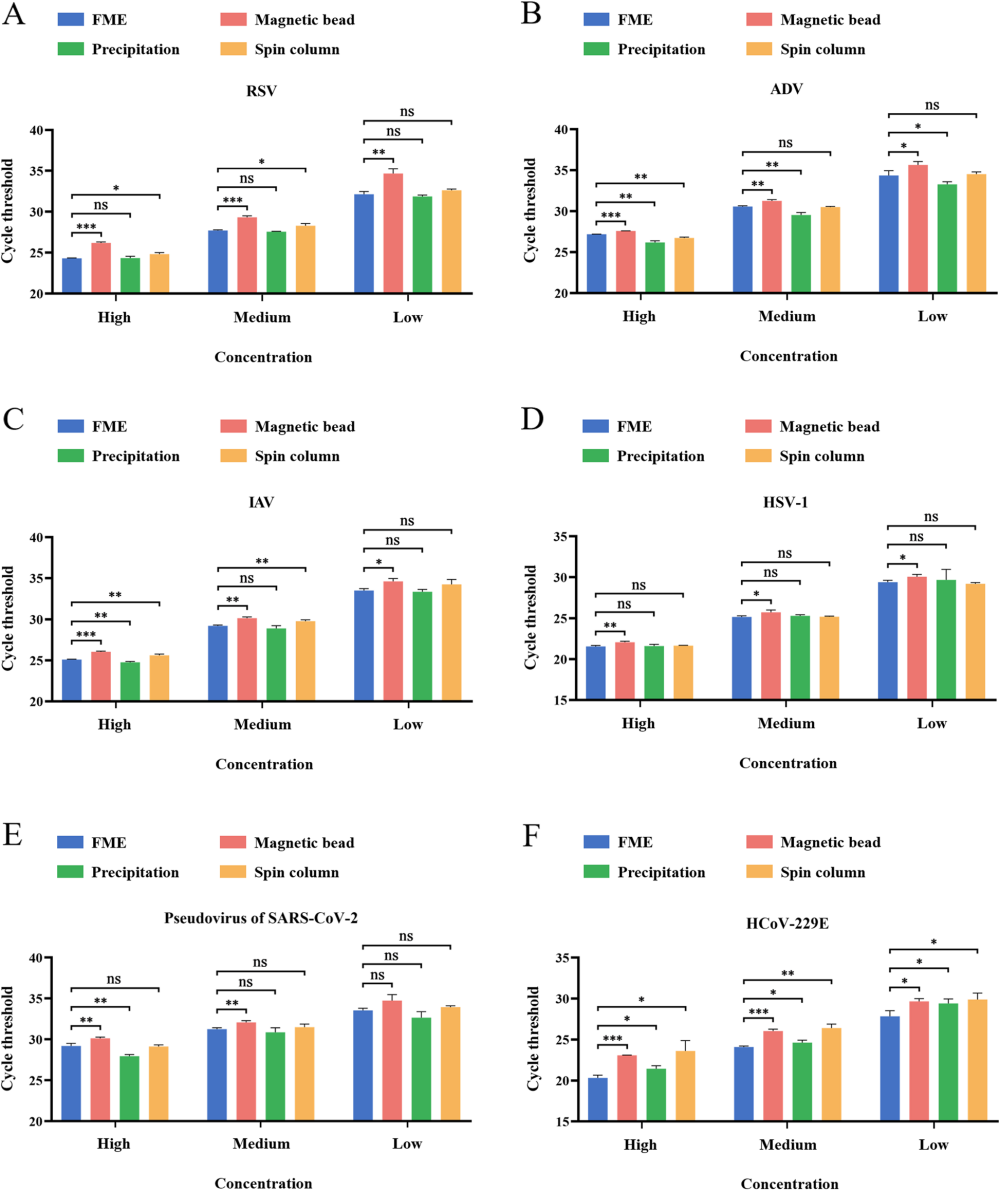
Effects of the FME on the extraction of multiple respiratory viruses. The efficiency of each method was evaluated by extracting high, medium, and low concentrations of respiratory viruses using the FME, magnetic bead (LC), spin column (TIANamp), and precipitation (GIRM) methods, followed by obtaining Ct values through qRT-PCR. **A** Effect of RSV nucleic acid extraction. **B** Effect of ADV nucleic acid extraction. **C** Effect of IAV nucleic acid extraction. **D** Effect of HSV-1 nucleic acid extraction. **E** Effect of SARS-CoV-2 pseudovirus nucleic acid extraction (*n* gene). **F** Effect of HCoV-229E nucleic acid extraction. Data are presented as means ± SD for three independent biological replicates. Statistical significance was calculated using *t* tests; ns *P* > 0.05, *P < 0.05, **P < 0.01, ***P < 0.001

To confirm the ability of automatic FME to extract actual samples, we selected several respiratory viruses commonly used in laboratories and compared the FME method with a representative magnetic bead method (LC), spin column method (TIANamp), and precipitation method (GIRM). The results showed that both the FME and the precipitation method exhibited the most effective extraction of samples with high, medium, and low concentrations of RSV, and the FME was superior to the spin column method and magnetic bead method (Fig. 4A). When extracting ADV, the traditional precipitation method was confirmed to be the most effective, followed by the FME and spin column, although the FME still outperformed the magnetic bead method at all concentrations (Fig. 4B). For IAV extraction, except for a slightly better performance of the precipitation method at the highest concentration compared with the FME, both the FME and precipitation method displayed optimal effects at all concentrations, surpassing other techniques (Fig. 4C). Regarding HSV-1 extraction, no significant difference was observed among the FME, precipitation, and spin column methods; however, all three were marginally more effective than the magnetic bead method (Fig. 4D). For high-concentration pseudovirus in a SARS-CoV-2 extraction, the precipitation method was the best, followed by the FME and spin column methods, while the magnetic bead method was the least effective. At a medium concentration, there was no significant difference among the FME, precipitation, and spin column methods, and all were superior to the magnetic bead method. At a low concentration, there was no significant difference among the four methods (Fig. 4E). In terms of high, medium, and low concentrations of HCoV-229E sample extractions, the FME exhibited the highest extraction effect, followed by the precipitation, magnetic bead, and spin column methods (Fig. 4F). This showed that FME achieved a superior or second-best outcome compared with commonly used nucleic acid extraction kits available on the market, regardless of whether the process involved extracting DNA or RNA viruses, or enveloped or non-enveloped viruses.

### Clinical performance of the FME

**Fig. 5:**
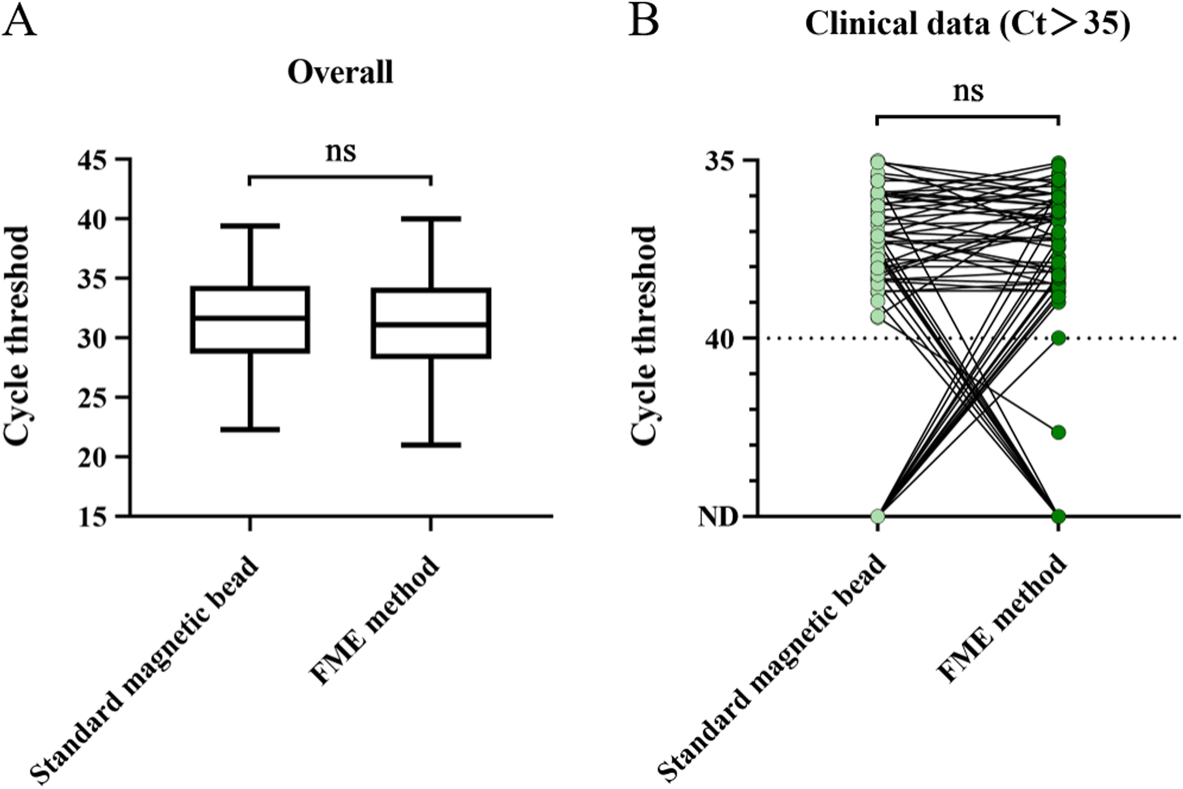
Distribution of Ct values for clinical specimens using the FME and standard magnetic bead method. **A** Distributions of Ct values obtained from IAV clinical specimens using the FME and standard magnetic bead extraction methods. **B** Distribution of Ct values for low-concentration specimens (Ct values > 35 for results of both tests). Lines within boxes represent medians. Upper and lower boundaries of boxes represent upper and lower quartiles, respectively. Bars represent minimum and maximum values. Dashed lines indicate the detection limit. ND, not detected. Statistical significance was calculated using *t* tests; ns *P* > 0.05

Frozen clinical specimens from 525 suspected IAV-infected patients were simultaneously extracted using both the standard magnetic bead method and the FME. The Ct values obtained by the standard magnetic bead method and FME were compared to evaluate the clinical performance of the FME. The detection performance of the FME for IAV, compared with that of the standard magnetic bead method, was as follows: sensitivity, 97.00% (323/333); specificity, 92.71% (178/192); positive predictive value, 95.85% (323/337); negative predictive value, 94.68% (178/188); and total coincidence rate (323+178)/525 = 95.43% (Table 2). The consistency between the two methods was good, with a Kappa statistic of 0.901 (*P* < 0.001). In addition, we analyzed the Ct value distribution of the clinical specimens. The average Ct values of IAV extracted by the standard magnetic bead method and FME were 31.49 and 31.16, respectively. There was no significant difference in the Ct value distribution between the two extraction methods. However, the Ct values obtained through the FME were broader in the upper and lower quartiles than those obtained by the magnetic bead method (Fig. 5A). Further analysis was conducted on specimens with Ct values > 35 to evaluate the limit of detection (LoD) of the FME assay in actual clinical samples. It was found that there was no significant difference in the Ct distribution between the FME and standard magnetic bead method in low-concentration specimens (Fig. 5B).

**Table 2:** Comparison of the FME and standard magnetic bead method for analyzing 525 clinical specimens with suspected IAV infection

FME	Standard magnetic bead method		Total
	Positive (+)	Negative (-)	
Positive (+)	323	14	337
Negative (-)	10	178	188
Total	333	192	525

We noticed that a total of 24 specimens had inconsistent results after being extracted using the FME and standard magnetic bead method. Among them, 14 cases were positive for FME detection and negative for standard magnetic bead method detection; 10 cases were negative for FME detection (including one case with a Ct value > 40, which was judged to be negative due to the interpretation criteria) and positive for standard magnetic bead method detection (Additional file 1: Table S3). All these inconsistent results had Ct values > 35, and 21 had Ct values > 37. Clearly, these specimens were borderline positive results. In addition to the difference in extraction efficiency, the detection performance of the qRT-PCR instrument on the LoD also affects the detection rate of the results. Overall, the effect of the FME on clinical specimen extraction was comparable to that of the standard magnetic bead method.

## Discussion

In recent years, the field of pathogen detection technology has witnessed an unprecedented pace of development. Compared with lateral flow immunochromatographic assays (LFIAs), enzyme-linked immunosorbent assays (ELISAs), plaque reduction neutralization tests (PRNTs), and other technologies, NAAT-based detection methods exhibit superior sensitivity, specificity, and speed of detection [22–24]. Among all NAATs, qRT-PCR is the most widely used and has emerged as the preferred method for detecting human pathogens [25, 26]. However, a major choke point of qRT-PCR diagnosis is that it relies on the extraction and purification of nucleic acids from samples, which is crucial for achieving optimal sensitivity, but also a relatively time-consuming and laborious process [27, 28]. It has been reported that extraction-free methods can serve as an alternative procedure, alleviating the supply bottleneck of extraction reagents [29]. However, these strategies are incapable of eliminating PCR inhibitors in the specimen, leading to a significant reduction in sensitivity when processing samples with low viral loads [30, 31]. Therefore, there is an urgent need for novel technology capable of rapidly extracting high-quality nucleic acid.

Glycerin functions as a humectant, solvent, plasticizer, adhesive, and binding agent. Despite its extensive utilization in the fields of food, cosmetics, medicine, and chemical synthesis, glycerin has not yet been employed as the primary raw material in nucleic acid purification reagents [32]. In this study, we demonstrated that glycerin, which is miscible with water in any proportion, can purify nucleic acids, and the higher the concentration of glycerin, the better the purification effect (Fig. 1B). Furthermore, after adding a certain proportion of EtOH to 50% glycerin, the effect of washing with 50% glycerin and 50% EtOH for a short duration was superior to that of thoroughly washing by the conventional magnetic bead method three times (Fig. 1C). However, we found that the elution of miscellaneous proteins using 50% glycerin and 50% EtOH primarily relied on the presence of EtOH (Fig. 1F and G); the specific role of glycerin in nucleic acid purification remains to be investigated.

To release nucleic acids from the sample, physical, chemical, or enzymatic methods are usually used to lyse samples [9]. In clinical laboratories, nucleic acid lysis commonly involves mixing the sample with a chemical lysis solution, subjecting it to high-temperature heating, and supplementing with proteinase K [21, 33]. In this study, we developed a chemical-based lysis solution AP, which exhibited a superior lysis effect compared with the majority of commercially available lysis reagents when combined with 50% glycerin and 50% EtOH for nucleic acid extraction (Fig. 2A). The FME, composed of AP, 50% glycerin and 50% EtOH, exhibited superior efficacy in RSV extraction compared with conventional methods (Fig. 3C). Moreover, the extracted RNA from AD293 cells demonstrated above-average levels of concentration and purity (Table 1). It is worth pointing out that commercially available kits have been modified to ensure consistent sample loading and elution volumes with FME, thus the above results may not have necessarily reflected the optimal efficiency of these kits; however, these findings highlight the advantages of FME in terms of speed and performance. We also explored the inhibition of the PCR reaction when there was residual AP lysis solution during elution when the concentration of each AP component reached a certain threshold. After the concentrations of GTC, sodium citrate, and sarkosyl reached a level that inhibited PCR amplification, an exponential increase in the Ct values resulted (Additional file 3: Figure S2). Subsequently, by diluting the FME elution solution 10-fold, we observed a sequential increase of approximately 3.4 in the detected Ct values (Additional file 4: Figure S3), indicating the absence of inhibitory residues within the elution. Although concentration and purity are important parameters for evaluating the quality of nucleic acid extraction, the ultimate indicator lies in its functionality in downstream applications [34].

Compared with other nucleic acid extraction techniques, the magnetic bead method offers several advantages, including unrestricted sample volumes, easy removal from sample suspensions, and suitability for large-scale automated operations. Moreover, various types of magnetic particles can be utilized for the separation and purification of DNA, RNA, and plasmid DNA [35, 36]. We equipped the FME with a small automatic nucleic acid extractor to automate the FME in the same manner as the manual operation in terms of extraction time, quality, and stability (Fig. 3F). We observed that the FME demonstrated superior or comparable efficacy in extracting respiratory viruses compared with magnetic bead, spin column, and precipitation methods (Fig. 4). We hypothesized that the disparity in extraction efficiency among different viruses was associated with the presence of a specific secondary structure and envelope. It should be noted that, in terms of time requirements, the magnetic bead method, when automated, required approximately 25–30 min, while both the spin column method and precipitation method, being manual processes, necessitated close to 1 h. Furthermore, both the spin column and precipitation methods require manual operations, which places high demands on the skills and proficiency of the operators. By contrast, the automatic FME not only extracted high-quality nucleic acid but also required less time with no technical requirements for personnel. In addition, our research showed that the FME had excellent clinical performance when analyzing specimens from individuals suspected to have IAV infection. The distribution of Ct values detected by FME in 525 clinical specimens was similar to that observed using the standard magnetic bead method (Fig. 5A), and there was no significant difference between the two methods in the analysis of low-concentration specimens close to the LoD (Fig. 5B), although FME identified more positive cases (Additional file 1: Table S3). In spite of this, it is essential to observe that the aforementioned clinical specimens refer to pharyngeal swabs, and other samples such as sputum, stool, and blood cannot be directly processed using the FME method. In the case of sputum and stool, pre-treatment is required prior to extraction, which typically takes a minimum of 30 min [37, 38]. Additionally, whole blood and plasma samples are highly viscous, and therefore require additional time for lysis, washing and magnetic bead magnetization. Consequently, when extracting viruses from complex samples, FME is not able to produce high-quality nucleic acid within 5 min. Collectively, these findings highlight the promising clinical applicability of FME for analyzing non-complex samples.

Recently, numerous innovative techniques for nucleic acid extraction have been reported, including the use of cellulose-based membranes to extract nucleic acid without the requirement of a separate elution step, enabling direct amplification from the membrane [39]. A polytetrafluoroethylene (PTFE)-based nucleic acid extraction system achieved a fully enclosed sample extraction process that seamlessly integrated with droplet digital PCR (ddPCR) [40]. Using an integrated microfluidic system, the process of sample extraction, enrichment, and detection was completed within a small chip [41]. Although these novel methodologies exhibited rapidity and compactness, their applicability remained confined to specific scenarios. To achieve widespread adoption in scientific research and clinical testing, challenges pertaining to automation, cost-effectiveness, and processing of intricate samples still need to be addressed. The results of qRT-PCR detection depend on a number of factors, including the specimen type, the timing of collection, the quality and quantity of viral RNA, the primer and probe design for viral RNA targets, the reagents and instruments used for detection, as well as the signals and cut-off values employed for result interpretation [42, 43]. Among them, obtaining high-quality nucleic acid is fundamental for accurate detection. However, when changing the RNA extraction kits from one type to other, particularly those based on different chemical components, the reliability of both the quantity and quality of extracted nucleic acid becomes uncertain [44, 45]. How to detect viruses with high Ct values and low loads stably, while avoiding false negatives, remains a challenging problem.

## Conclusion

In summary, this study demonstrates that glycerin served as an effective washing solution for nucleic acid purification and presented a novel nucleic acid extraction technology based on glycerin with a remarkable processing time of only 5 min. The developed technology exhibited notable features, including rapidity, cost-effectiveness, automation, and high-quality yields of extracted nucleic acids. Consequently, this study significantly contributes to the reduction of sample pre-processing time in NAATs and lays a foundation for the realization of rapid molecular diagnosis.

### Supplementary Information

Additional file 1. 

Additional file 2: Figure S1. The LC exhibits an extraction efficiency similar to the magnetic bead method. A and B RSV and ADV nucleic acid were extracted using LC, Baypure, and HR magnetic bead extraction kits, and qRT-PCR Ct values were used to assess the efficiency of each method for extracting nucleic acid. A: Effect of RSV nucleic acid extraction. B: Effect of ADV nucleic acid extraction. Data are presented as means± SD for three independent biological replicates. Statistical significance was calculated using *t* tests; ns *P* > 0.05, *P < 0.05, **P < 0.01.

Additional file 3: Figure S2. Effect of residual AP lysis solution on PCR amplification. RSV nucleic acid was extracted at a high concentration by the FME. A and B In the RSV reaction system, 4 μL of RSV nucleic acid was added, along with an additional 1 μL each of DEPC H_2_O, 25 mM sodium citrate, 20 mM sarkosyl, 2.5% PEG 6000, 1 M DTT, and 4 M GTC, IPA, or AP lysis solution. qRT-PCR Ct values were used to assess the effect of the residual AP lysis solution components on amplification. C and D 4 μL of RSV nucleic acid was added, followed by an additional 1 μL of sodium citrate diluted in a twofold gradient. Ct values were used to assess the effect of residual sodium citrate on PCR amplification. E and F 4 μL of RSV nucleic acid was added, followed by an additional 1 μL of sarkosyl diluted in a twofold gradient. Ct values were used to assess the effect of residual sarkosyl on PCR amplification. G and H 4 μL of RSV nucleic acid was added, followed by an additional 1 μL of GTC in a twofold gradient. Ct values were used to assess the effect of GTC on PCR amplification. Solid lines indicate the median, and dashed lines indicate the detection limit. Data are presented as means ± SD for three or four independent biological replicates.

Additional file 4: Figure S3. The Ct values of the eluted nucleic acid were determined following a 10-fold gradient dilution. High concentrations of RSV were extracted by the FME, and the nucleic acid in the elution was sequentially diluted using a 10-fold gradient with DEPC H_2_O. The Ct values were determined by qRT-PCR for each dilution gradient, with each measurement repeated three times. A Amplification curve. B Linear regression curve, *R*^*2*^= 0.9993.

Additional file 5. 

Additional file 6. 

## Data Availability

All data generated or analyzed during this study are included in this published article and its supplementary information files.

## Abbreviations

RSV: Respiratory syncytial virus
ADV: Adenovirus
SARS-CoV-2: Severe acute respiratory syndrome coronavirus 2
IAV: Influenza A virus
NAAT: Nucleic acid amplification test
qRT-PCR: Quantitative real-time polymerase chain reaction
IATs: Isothermal amplification technologies
NGS: Next-generation sequencing
GTC: Guanidine thiocyanate
RNase P: Ribonuclease P
DMEM: Dulbecco’s modified Eagle medium
FBS: Fetal bovine serum
HSV-1: Herpes simplex virus 1
HCoV-229E: Human coronavirus 229E
FME: Five-minute nucleic acid extraction
CFDA: China Food and Drug Administration
Sodium citrate: Sodium citrate tribasic dihydrate
Sarkosyl: Sodium lauroyl-sarcosine
DTT: Dithiothreitol
PEG 6000: Polyethylene glycol 6000
IPA: Isopropyl alcohol
EtOH: Ethanol
EDTA: Ethylene diamine tetraacetic acid
LC: LemnisCare
Ct: Cycle threshold
BSA: Bovine serum albumin
AP: A Plus lysis solution
Rn: Normalized reporter
LoD: Limit of detection
LFIAs: Lateral flow immunochromatographic assays
ELISAs: Enzyme-linked immunosorbent assays
PRNTs: Plaque reduction neutralization tests
PTFE: Polytetrafluoroethylene
ddPCR: Droplet digital PCR
DEPC: Diethyl pyrocarbonate
PBS: Phosphate-buffered saline
