# Supplementary material for: Development and validation of a rapid five-minute nucleic acid extraction method for respiratory viruses
Source: Virol J. 2024 Aug 18;21:189. doi: 10.1186/s12985-024-02381-3 (PMC11331601; doi:10.1186/s12985-024-02381-3)
Supplement: Supplementary file 1 — Additional file 1. [file 12985_2024_2381_MOESM1_ESM.docx]

**Table S1.** Primers used in this study

| Primer name | Description | Primer sequence (5’–3’) |
| --- | --- | --- |
| HSV-1-F | Real-time PCR primer, forward | TGACTCAGGGCTCTACCCTC |
| HSV-1-R | Real-time PCR primer, reverse | CTCGACCACGGTTCGAAAGA |
| HCoV-229E-F | Real-time PCR primer, forward | GCCTTGGTGCCGTAGATGAA |
| HCoV-229E-R | Real-time PCR primer, reverse | TGTCAAGCCAAAGCAAGGGA |

**Table S2.** Automation program for the FME

| Step | Position | Waiting time (s) | Mixing time (s) | Volume (μL) | Magnetic setting | | Temperature （°C） |
| --- | --- | --- | --- | --- | --- | --- | --- |
|  |  |  |  |  | Time (s) | No. of cycles |  |
| 1 Magnetic bead transfer | 2 | 0 | 0 | 400 | 10 | 1 | 25 |
| 2 Lysis | 1 | 0 | 60 | 500 | 30 | 1 | 25 |
| 3 Washing | 3 | 0 | 60 | 300 | 60 | 4 | 25 |
| 4 Elution | 6 | 0 | 30 | 100 | 30 | 1 | 56 |
| 5 Magnetic bead transfer | 2 | 0 | 5 | 400 | 0 | 1 | 25 |

Abbreviations: FME, five-minute nucleic acid extraction; No., number.

**Table S3.** Clinical data of IAV specimens

| Serial No. | Specimen | Standard magnetic bead method  (Ct values) | | FME  (Ct values) | |
| --- | --- | --- | --- | --- | --- |
|  |  | IAV | RNase P | IAV | RNase P |
| 1 | 20003 | - | 33.22 | - | 34.19 |
| 2 | 20004 | 34.90 | 34.07 | 35.42 | 34.85 |
| 3 | 20005 | - | 29.90 | - | 30.44 |
| 4 | 20006 | 32.46 | 29.65 | 31.04 | 30.56 |
| 5 | 20007 | - | 31.50 | - | 31.54 |
| 6 | 20008 | - | 34.59 | 39.99 | 35.35 |
| 7 | 20009 | 39.41 | 29.40 | 42.64 | 29.69 |
| 8 | 20010 | - | 30.53 | - | 31.04 |
| 9 | 20011 | - | 30.98 | - | 31.41 |
| 10 | 20012 | - | 30.83 | - | 30.83 |
| 11 | 20013 | 37.71 | 28.61 | - | 29.18 |
| 12 | 20014 | 38.33 | 30.23 | 38.46 | 30.70 |
| 13 | 20015 | 35.04 | 30.85 | 34.90 | 33.72 |
| 14 | 20016 | 38.38 | 32.14 | 38.70 | 32.75 |
| 15 | 20017 | - | 27.16 | - | 26.03 |
| 16 | 20018 | - | 31.55 | - | 33.78 |
| 17 | 20019 | - | 25.42 | - | 24.62 |
| 18 | 20020 | - | 31.23 | - | 32.07 |
| 19 | 20021 | - | 30.15 | - | 29.63 |
| 20 | 20022 | 35.72 | 30.85 | 34.78 | 30.29 |
| 21 | 20023 | 36.41 | 31.55 | 35.93 | 31.27 |
| 22 | 20024 | 33.95 | 30.40 | 34.14 | 30.52 |
| 23 | 20025 | - | 28.84 | - | 28.77 |
| 24 | 20026 | 32.53 | 31.47 | 30.70 | 30.16 |
| 25 | 20027 | 38.29 | 31.89 | 36.64 | 33.08 |
| 26 | 20028 | - | 30.74 | - | 32.05 |
| 27 | 20029 | - | 27.49 | - | 27.94 |
| 28 | 20030 | - | 27.70 | - | 27.16 |
| 29 | 20031 | - | 29.91 | 38.74 | 30.01 |
| 30 | 20032 | 27.50 | 30.87 | 26.33 | 30.49 |
| 31 | 20033 | - | 28.10 | - | 26.56 |
| 32 | 20034 | - | 29.86 | - | 29.41 |
| 33 | 20035 | 31.25 | 30.02 | 30.94 | 31.25 |
| 34 | 20036 | 33.52 | 29.09 | 33.73 | 30.15 |
| 35 | 20037 | 38.70 | 29.42 | 38.44 | 30.47 |
| 36 | 20038 | 35.04 | 30.95 | 35.97 | 31.61 |
| 37 | 20039 | - | 29.34 | - | 30.72 |
| 38 | 20040 | - | 30.56 | - | 32.65 |
| 39 | 20041 | 32.87 | 30.22 | 32.39 | 31.78 |
| 40 | 20042 | 31.71 | 31.57 | 31.67 | 32.09 |
| 41 | 20043 | 33.41 | 30.53 | 34.36 | 32.35 |
| 42 | 20044 | 35.98 | 30.36 | 36.63 | 31.26 |
| 43 | 20045 | - | 29.02 | - | 29.36 |
| 44 | 20046 | - | 28.87 | - | 29.48 |
| 45 | 20047 | 30.34 | 31.71 | 30.25 | 33.30 |
| 46 | 20048 | - | 29.40 | - | 29.12 |
| 47 | 20049 | - | 30.00 | - | 31.42 |
| 48 | 20050 | - | 29.68 | - | 32.89 |
| 49 | 20051 | - | 29.61 | - | 30.29 |
| 50 | 20052 | - | 28.49 | - | 30.85 |
| 51 | 20053 | 29.89 | 30.18 | 29.31 | 29.79 |
| 52 | 20054 | 28.16 | 30.05 | 27.25 | 29.24 |
| 53 | 20055 | 31.78 | 29.21 | 32.13 | 28.15 |
| 54 | 20056 | 27.87 | 30.71 | 27.65 | 32.44 |
| 55 | 20057 | - | 31.08 | - | 30.47 |
| 56 | 20058 | - | 29.49 | - | 30.61 |
| 57 | 20059 | - | 28.39 | - | 29.96 |
| 58 | 20060 | - | 28.36 | - | 29.88 |
| 59 | 20061 | - | 31.66 | - | 31.14 |
| 60 | 20062 | 25.68 | 29.81 | 26.92 | 33.50 |
| 61 | 20063 | 27.15 | 29.85 | 25.90 | 29.10 |
| 62 | 20064 | 32.46 | 29.26 | 33.32 | 31.01 |
| 63 | 20065 | - | 33.11 | - | 33.25 |
| 64 | 20066 | 32.33 | 32.06 | 32.33 | 32.56 |
| 65 | 20067 | 36.22 | 30.04 | 36.71 | 30.69 |
| 66 | 20068 | 33.27 | 30.01 | 33.39 | 29.37 |
| 67 | 20069 | 32.48 | 31.30 | 31.71 | 31.45 |
| 68 | 20070 | - | 30.35 | - | 29.50 |
| 69 | 20071 | 34.35 | 31.20 | 34.66 | 31.89 |
| 70 | 20072 | - | 30.89 | - | 32.85 |
| 71 | 20073 | 35.60 | 30.17 | - | 30.83 |
| 72 | 20074 | 31.15 | 32.83 | 30.82 | 33.57 |
| 73 | 20075 | - | 31.85 | - | 33.55 |
| 74 | 20076 | 31.52 | 31.26 | 33.37 | 31.92 |
| 75 | 20077 | - | 32.69 | - | 31.27 |
| 76 | 20078 | 33.49 | 30.44 | 34.00 | 32.09 |
| 77 | 20079 | 33.36 | 26.97 | 32.68 | 26.80 |
| 78 | 20080 | 25.60 | 32.01 | 25.51 | 33.23 |
| 79 | 20081 | 31.76 | 31.05 | 31.66 | 32.97 |
| 80 | 20082 | 28.34 | 30.32 | 30.00 | 29.76 |
| 81 | 20083 | 32.93 | 31.41 | 33.96 | 31.10 |
| 82 | 20084 | 32.64 | 28.51 | 32.33 | 28.18 |
| 83 | 20085 | - | 32.02 | - | 32.28 |
| 84 | 20086 | - | 33.32 | - | 33.48 |
| 85 | 20087 | 34.33 | 29.85 | 33.20 | 28.01 |
| 86 | 20088 | - | 27.84 | - | 28.34 |
| 87 | 20089 | 36.02 | 28.60 | 35.08 | 29.48 |
| 88 | 20090 | 34.11 | 30.76 | 37.31 | 31.34 |
| 89 | 20091 | - | 27.92 | - | 28.48 |
| 90 | 20092 | 33.65 | 31.17 | 33.65 | 32.66 |
| 91 | 20093 | 33.51 | 31.37 | 33.93 | 32.93 |
| 92 | 20094 | 30.61 | 31.06 | 30.64 | 30.73 |
| 93 | 20095 | - | 32.11 | - | 32.06 |
| 94 | 20096 | 32.93 | 29.17 | 31.83 | 29.73 |
| 95 | 20097 | - | 26.35 | - | 26.36 |
| 96 | 20098 | 25.32 | 28.36 | 24.85 | 27.87 |
| 97 | 20099 | 28.41 | 30.50 | 27.34 | 31.13 |
| 98 | 20100 | 30.17 | 30.77 | 29.15 | 30.41 |
| 99 | 20101 | 33.02 | 31.74 | 32.67 | 32.63 |
| 100 | 20102 | - | 30.46 | - | 30.21 |
| 101 | 20103 | 32.84 | 29.25 | 32.19 | 29.13 |
| 102 | 20104 | - | 29.98 | - | 30.63 |
| 103 | 20105 | 27.85 | 31.24 | 30.41 | 33.77 |
| 104 | 20106 | 29.25 | 29.89 | 29.21 | 30.63 |
| 105 | 20107 | 34.45 | 29.02 | 33.90 | 30.08 |
| 106 | 20108 | - | 31.63 | - | 34.04 |
| 107 | 20109 | 29.17 | 29.27 | 28.94 | 30.69 |
| 108 | 20110 | 26.62 | 29.48 | 26.82 | 31.15 |
| 109 | 20111 | 31.17 | 30.20 | 31.31 | 32.02 |
| 110 | 20112 | 33.71 | 30.46 | 37.77 | 32.70 |
| 111 | 20113 | - | 30.01 | - | 31.11 |
| 112 | 20114 | 24.26 | 30.27 | 24.85 | 30.71 |
| 113 | 20115 | 29.89 | 32.90 | 29.20 | 32.80 |
| 114 | 20116 | 38.67 | 33.28 | 38.66 | 33.73 |
| 115 | 20117 | 31.96 | 32.64 | 34.13 | 32.90 |
| 116 | 20118 | 31.66 | 31.52 | 34.46 | 31.96 |
| 117 | 20119 | 29.31 | 30.92 | 30.27 | 30.93 |
| 118 | 20120 | 28.88 | 31.11 | 29.10 | 34.37 |
| 119 | 20121 | 28.22 | 26.95 | 30.24 | 27.45 |
| 120 | 20122 | 38.36 | 28.17 | 38.11 | 28.95 |
| 121 | 20123 | - | 29.01 | - | 29.40 |
| 122 | 20124 | 32.66 | 28.85 | 33.23 | 30.36 |
| 123 | 20125 | 31.18 | 31.36 | 31.73 | 36.12 |
| 124 | 20126 | 26.13 | 30.08 | 25.92 | 29.77 |
| 125 | 20127 | 22.59 | 30.15 | 23.33 | 30.13 |
| 126 | 20128 | - | 27.27 | - | 26.58 |
| 127 | 20129 | - | 28.89 | - | 27.86 |
| 128 | 20130 | - | 33.24 | 38.55 | 33.09 |
| 129 | 20131 | - | 29.61 | - | 29.32 |
| 130 | 20132 | 28.68 | 30.28 | 28.62 | 29.48 |
| 131 | 20133 | 30.32 | 31.38 | 31.05 | 30.04 |
| 132 | 20134 | 29.93 | 29.26 | 29.92 | 29.76 |
| 133 | 20135 | - | 28.31 | - | 26.68 |
| 134 | 20136 | 28.15 | 28.18 | 27.74 | 28.55 |
| 135 | 20137 | - | 30.13 | - | 32.53 |
| 136 | 20138 | 37.53 | 30.71 | - | 30.78 |
| 137 | 20139 | 30.62 | 32.45 | 30.35 | 33.26 |
| 138 | 20140 | 37.35 | 28.78 | 37.25 | 28.66 |
| 139 | 20141 | 32.21 | 30.07 | 32.43 | 32.25 |
| 140 | 20142 | 32.99 | 32.64 | 32.59 | 32.95 |
| 141 | 20143 | 34.55 | 30.29 | 33.80 | 29.56 |
| 142 | 20144 | 27.19 | 29.81 | 26.01 | 29.76 |
| 143 | 20145 | 27.30 | 30.78 | 26.14 | 31.01 |
| 144 | 20146 | 29.28 | 27.34 | 28.32 | 27.65 |
| 145 | 20147 | 34.05 | 31.10 | 32.62 | 32.00 |
| 146 | 20148 | 29.50 | 31.64 | 29.45 | 33.25 |
| 147 | 20149 | - | 32.45 | - | 33.90 |
| 148 | 20150 | 25.83 | 28.74 | 25.04 | 28.39 |
| 149 | 20151 | 28.50 | 31.70 | 27.59 | 32.04 |
| 150 | 20152 | 37.23 | 29.53 | 38.51 | 31.78 |
| 151 | 20153 | 28.82 | 30.28 | 28.66 | 33.32 |
| 152 | 20154 | 33.07 | 29.71 | 33.22 | 32.66 |
| 153 | 20155 | 35.90 | 30.98 | 35.73 | 31.73 |
| 154 | 20156 | 32.73 | 30.56 | 33.29 | 33.55 |
| 155 | 20157 | 28.24 | 28.00 | 28.05 | 28.05 |
| 156 | 20158 | 30.69 | 28.98 | 30.62 | 29.33 |
| 157 | 20159 | - | 31.22 | - | 31.78 |
| 158 | 20160 | 34.48 | 29.96 | 34.54 | 30.17 |
| 159 | 20161 | 31.68 | 32.16 | 29.85 | 31.39 |
| 160 | 20162 | - | 32.00 | - | 32.33 |
| 161 | 20163 | - | 32.46 | - | 31.73 |
| 162 | 20164 | 39.38 | 32.02 | 36.10 | 31.44 |
| 163 | 20165 | 35.77 | 28.19 | 34.11 | 26.36 |
| 164 | 20166 | - | 31.38 | - | 30.32 |
| 165 | 20167 | 34.04 | 32.00 | 31.57 | 31.59 |
| 166 | 20168 | - | 34.39 | - | 36.30 |
| 167 | 20169 | 35.12 | 28.01 | 34.02 | 27.86 |
| 168 | 20170 | 35.46 | 29.36 | 34.21 | 29.72 |
| 169 | 20171 | 33.74 | 30.09 | 33.73 | 30.28 |
| 170 | 20172 | - | 29.66 | - | 29.34 |
| 171 | 20173 | - | 31.84 | - | 31.31 |
| 172 | 20174 | 35.70 | 29.10 | 34.96 | 29.53 |
| 173 | 20175 | 32.54 | 32.80 | 31.72 | 32.10 |
| 174 | 20176 | 33.08 | 30.36 | 33.31 | 31.02 |
| 175 | 20177 | 34.84 | 30.48 | 34.60 | 30.08 |
| 176 | 20178 | 27.96 | 28.89 | 27.79 | 29.29 |
| 177 | 20179 | 37.60 | 30.10 | - | 30.48 |
| 178 | 20180 | - | 31.89 | - | 32.44 |
| 179 | 20181 | 31.49 | 31.28 | 31.13 | 33.05 |
| 180 | 20182 | - | 31.47 | - | 31.83 |
| 181 | 20183 | 34.78 | 29.59 | 34.78 | 29.31 |
| 182 | 20184 | 29.13 | 29.01 | 29.04 | 29.97 |
| 183 | 20185 | 24.34 | 29.91 | 24.09 | 30.74 |
| 184 | 20186 | 26.30 | 27.74 | 26.46 | 28.90 |
| 185 | 20187 | 29.56 | 29.92 | 30.00 | 30.56 |
| 186 | 20188 | 31.86 | 29.94 | 32.08 | 32.52 |
| 187 | 20189 | 29.07 | 29.60 | 29.18 | 29.60 |
| 188 | 20190 | 28.78 | 30.09 | 29.11 | 29.34 |
| 189 | 20191 | 31.68 | 30.95 | 32.90 | 31.33 |
| 190 | 20192 | 23.04 | 27.87 | 22.81 | 29.08 |
| 191 | 20193 | 30.86 | 27.57 | 30.12 | 26.69 |
| 192 | 20194 | 27.96 | 25.74 | 27.20 | 25.34 |
| 193 | 20195 | 22.99 | 29.03 | 24.12 | 29.48 |
| 194 | 20196 | 35.46 | 31.01 | 36.21 | 31.02 |
| 195 | 20197 | 34.42 | 30.24 | 34.39 | 30.81 |
| 196 | 20198 | - | 30.80 | - | 30.83 |
| 197 | 20199 | 22.26 | 28.39 | 22.78 | 27.90 |
| 198 | 20200 | 31.38 | 29.23 | 31.08 | 29.11 |
| 199 | 20201 | 30.49 | 29.14 | 30.64 | 30.03 |
| 200 | 20202 | 28.64 | 28.29 | 28.47 | 29.25 |
| 201 | 20203 | 30.76 | 28.01 | 30.01 | 29.03 |
| 202 | 20204 | 25.90 | 29.63 | 25.14 | 30.45 |
| 203 | 20205 | - | 25.67 | - | 25.87 |
| 204 | 20206 | 31.96 | 27.25 | 31.52 | 26.41 |
| 205 | 20207 | 33.96 | 27.72 | 34.13 | 27.43 |
| 206 | 20208 | 33.61 | 30.81 | 35.15 | 32.14 |
| 207 | 20209 | - | 31.10 | 37.71 | 32.03 |
| 208 | 20210 | - | 28.36 | - | 27.79 |
| 209 | 20211 | 36.55 | 31.67 | 37.45 | 30.73 |
| 210 | 20212 | - | 26.29 | - | 25.83 |
| 211 | 20213 | 33.33 | 30.04 | 34.18 | 29.62 |
| 212 | 20214 | - | 29.28 | - | 30.62 |
| 213 | 20215 | 27.47 | 30.78 | 28.02 | 31.47 |
| 214 | 20216 | 35.36 | 26.36 | 35.95 | 26.45 |
| 215 | 20217 | 30.61 | 29.17 | 31.42 | 28.83 |
| 216 | 20218 | 34.03 | 32.08 | 34.46 | 34.23 |
| 217 | 20219 | 34.21 | 30.65 | 35.42 | 31.87 |
| 218 | 20220 | 32.81 | 29.70 | 34.21 | 29.82 |
| 219 | 20221 | 32.45 | 28.29 | 33.24 | 27.44 |
| 220 | 20222 | 36.21 | 32.25 | 35.92 | 33.14 |
| 221 | 20223 | 32.98 | 29.27 | 33.79 | 29.14 |
| 222 | 20224 | - | 30.87 | - | 30.93 |
| 223 | 20225 | 30.99 | 33.19 | 30.61 | 33.27 |
| 224 | 20226 | - | 33.92 | - | 33.93 |
| 225 | 20227 | 35.80 | 32.16 | 33.79 | 31.08 |
| 226 | 20228 | - | 29.56 | 38.31 | 30.29 |
| 227 | 20229 | 28.92 | 31.02 | 28.57 | 31.21 |
| 228 | 20230 | 36.80 | 30.88 | 35.38 | 31.43 |
| 229 | 20231 | - | 33.12 | - | 32.78 |
| 230 | 20232 | - | 28.94 | - | 29.68 |
| 231 | 20233 | - | 25.02 | - | 25.42 |
| 232 | 20234 | 30.35 | 31.68 | 30.87 | 32.80 |
| 233 | 20235 | 33.34 | 28.51 | 32.71 | 29.16 |
| 234 | 20236 | 32.91 | 28.51 | 32.37 | 29.54 |
| 235 | 20237 | - | 31.31 | - | 31.92 |
| 236 | 20238 | 35.51 | 31.36 | 34.81 | 32.20 |
| 237 | 20239 | - | 29.00 | - | 28.59 |
| 238 | 20240 | 35.02 | 27.36 | 37.19 | 26.48 |
| 239 | 20241 | 35.06 | 29.79 | 35.64 | 29.51 |
| 240 | 20242 | 29.78 | 31.01 | 30.43 | 30.64 |
| 241 | 20243 | - | 31.29 | - | 28.42 |
| 242 | 20244 | 31.21 | 30.60 | 32.93 | 31.32 |
| 243 | 20245 | 30.07 | 29.95 | 30.63 | 30.63 |
| 244 | 20246 | - | 27.25 | - | 27.20 |
| 245 | 20247 | 33.24 | 30.61 | 33.08 | 31.05 |
| 246 | 20248 | 35.86 | 29.72 | 36.26 | 30.37 |
| 247 | 20249 | 26.84 | 29.64 | 26.14 | 29.75 |
| 248 | 20250 | 35.91 | 33.24 | 36.70 | 33.17 |
| 249 | 20251 | 25.22 | 28.54 | 25.98 | 28.33 |
| 250 | 20252 | 24.47 | 31.01 | 25.42 | 30.38 |
| 251 | 20253 | 23.97 | 29.10 | 24.19 | 30.99 |
| 252 | 20254 | 37.28 | 30.15 | - | 29.10 |
| 253 | 20255 | 25.69 | 28.84 | 27.04 | 27.47 |
| 254 | 20256 | 28.63 | 28.69 | 28.60 | 28.68 |
| 255 | 20257 | 24.32 | 30.65 | 24.07 | 29.76 |
| 256 | 20258 | 28.44 | 27.77 | 27.16 | 25.81 |
| 257 | 20259 | 34.79 | 30.09 | 35.29 | 29.54 |
| 258 | 20260 | 28.84 | 29.15 | 28.56 | 28.88 |
| 259 | 20261 | 34.06 | 32.05 | 34.07 | 31.18 |
| 260 | 20262 | 37.08 | 29.34 | 38.05 | 28.10 |
| 261 | 20263 | 25.19 | 30.05 | 24.67 | 29.17 |
| 262 | 20264 | 24.36 | 29.83 | 24.51 | 29.40 |
| 263 | 20265 | 23.09 | 30.92 | 20.96 | 30.04 |
| 264 | 20266 | 28.10 | 31.16 | 28.12 | 30.63 |
| 265 | 20267 | 34.14 | 29.93 | 34.78 | 29.60 |
| 266 | 20268 | 26.59 | 29.84 | 26.39 | 29.31 |
| 267 | 20269 | - | 29.81 | 38.99 | 30.13 |
| 268 | 20270 | 23.31 | 28.66 | 22.59 | 28.52 |
| 269 | 20271 | 29.15 | 30.07 | 28.26 | 30.51 |
| 270 | 20272 | 37.03 | 29.06 | - | 28.57 |
| 271 | 20273 | - | 31.12 | 38.84 | 30.39 |
| 272 | 20274 | 30.53 | 30.58 | 30.01 | 30.70 |
| 273 | 20275 | 30.42 | 31.07 | 29.98 | 31.32 |
| 274 | 20276 | 25.13 | 28.59 | 24.37 | 28.48 |
| 275 | 20277 | 32.73 | 32.60 | 32.40 | 32.98 |
| 276 | 20278 | 28.87 | 30.29 | 27.97 | 29.83 |
| 277 | 20279 | 28.11 | 31.15 | 27.28 | 30.41 |
| 278 | 20280 | - | 31.43 | 37.19 | 31.96 |
| 279 | 20281 | 24.49 | 29.19 | 24.73 | 28.92 |
| 280 | 20282 | 26.73 | 28.85 | 27.00 | 28.59 |
| 281 | 20283 | 28.60 | 30.97 | 28.57 | 31.55 |
| 282 | 20284 | 34.30 | 28.89 | 33.40 | 28.12 |
| 283 | 20285 | - | 28.52 | - | 27.28 |
| 284 | 20286 | - | 29.37 | - | 28.30 |
| 285 | 20287 | 32.40 | 30.35 | 32.94 | 29.10 |
| 286 | 20288 | 26.58 | 27.72 | 25.42 | 27.25 |
| 287 | 20289 | 32.97 | 29.37 | 32.13 | 31.17 |
| 288 | 20290 | - | 29.40 | - | 30.44 |
| 289 | 20291 | 30.40 | 28.24 | 29.54 | 27.96 |
| 290 | 20292 | - | 31.95 | - | 33.43 |
| 291 | 20293 | 35.89 | 30.28 | 34.11 | 30.28 |
| 292 | 20294 | 30.31 | 30.07 | 29.47 | 32.15 |
| 293 | 20295 | 29.37 | 30.38 | 28.23 | 30.22 |
| 294 | 20296 | 31.71 | 29.24 | 31.26 | 28.88 |
| 295 | 20297 | 27.46 | 27.30 | 23.98 | 27.07 |
| 296 | 20298 | - | 27.68 | - | 28.20 |
| 297 | 20299 | 23.98 | 28.92 | 23.23 | 28.45 |
| 298 | 20300 | - | 28.71 | - | 28.64 |
| 299 | 20301 | 32.83 | 27.36 | 32.53 | 27.72 |
| 300 | 20302 | - | 32.04 | - | 32.34 |
| 301 | 20303 | - | 29.07 | - | 30.20 |
| 302 | 20304 | - | 28.89 | 35.81 | 27.47 |
| 303 | 20305 | 29.55 | 28.29 | 29.08 | 27.02 |
| 304 | 20306 | 30.73 | 30.92 | 30.30 | 29.83 |
| 305 | 20307 | - | 29.01 | - | 28.50 |
| 306 | 20308 | 29.95 | 28.16 | 28.72 | 27.69 |
| 307 | 20309 | 29.39 | 27.90 | 28.54 | 28.00 |
| 308 | 20310 | 28.23 | 29.87 | 26.81 | 30.17 |
| 309 | 20311 | - | 31.63 | - | 32.21 |
| 310 | 20312 | 31.45 | 31.33 | 31.04 | 33.37 |
| 311 | 20313 | 26.84 | 25.90 | 25.35 | 25.25 |
| 312 | 20314 | 34.95 | 30.57 | 34.09 | 33.71 |
| 313 | 20315 | - | 29.88 | - | 31.04 |
| 314 | 20316 | - | 29.67 | - | 30.32 |
| 315 | 20317 | 32.09 | 28.01 | 31.73 | 28.89 |
| 316 | 20318 | - | 30.16 | - | 28.63 |
| 317 | 20319 | 25.13 | 30.69 | 24.09 | 36.28 |
| 318 | 20320 | 32.34 | 29.15 | 31.38 | 30.31 |
| 319 | 20321 | - | 29.10 | - | 30.01 |
| 320 | 20322 | - | 31.69 | - | 33.84 |
| 321 | 20323 | - | 29.32 | - | 30.26 |
| 322 | 20324 | - | 30.99 | - | 28.66 |
| 323 | 20325 | - | 30.15 | - | 28.22 |
| 324 | 20326 | 32.57 | 29.22 | 31.97 | 28.46 |
| 325 | 20327 | 27.49 | 29.01 | 26.10 | 29.37 |
| 326 | 20328 | 33.10 | 30.27 | 32.11 | 32.04 |
| 327 | 20329 | - | 28.33 | - | 29.95 |
| 328 | 20330 | 30.25 | 30.34 | 29.30 | 32.48 |
| 329 | 20331 | 38.00 | 30.44 | 37.98 | 30.97 |
| 330 | 20332 | 27.34 | 30.80 | 26.27 | 33.75 |
| 331 | 20333 | 31.20 | 29.55 | 29.97 | 28.85 |
| 332 | 20334 | 29.76 | 28.18 | 28.85 | 28.03 |
| 333 | 20335 | - | 29.18 | - | 30.64 |
| 334 | 20336 | 29.49 | 28.15 | 29.26 | 28.32 |
| 335 | 20337 | 30.25 | 30.66 | 30.08 | 30.36 |
| 336 | 20338 | 32.24 | 30.67 | 33.37 | 30.73 |
| 337 | 20339 | 34.19 | 28.62 | 36.26 | 28.52 |
| 338 | 20340 | 28.47 | 27.16 | 28.31 | 26.69 |
| 339 | 20341 | 24.01 | 27.60 | 25.12 | 27.06 |
| 340 | 20342 | 30.06 | 29.81 | 29.12 | 30.80 |
| 341 | 20343 | 27.54 | 27.89 | 26.72 | 26.80 |
| 342 | 20344 | - | 30.11 | 38.27 | 29.28 |
| 343 | 20345 | 34.38 | 31.30 | 33.75 | 32.53 |
| 344 | 20346 | 27.01 | 31.41 | 25.09 | 31.90 |
| 345 | 20347 | 25.58 | 29.03 | 24.93 | 29.07 |
| 346 | 20348 | 37.05 | 29.02 | 38.19 | 30.06 |
| 347 | 20349 | 36.80 | 29.19 | 36.74 | 29.58 |
| 348 | 20350 | 30.10 | 30.13 | 29.22 | 30.57 |
| 349 | 20351 | 31.44 | 29.19 | 30.51 | 29.10 |
| 350 | 20352 | 29.39 | 28.10 | 28.72 | 28.14 |
| 351 | 20353 | 36.29 | 31.75 | 35.16 | 31.71 |
| 352 | 20354 | 30.46 | 30.86 | 27.55 | 29.52 |
| 353 | 20355 | - | 29.11 | - | 28.67 |
| 354 | 20356 | 29.98 | 28.01 | 28.87 | 27.78 |
| 355 | 20357 | 38.10 | 28.93 | - | 29.19 |
| 356 | 20358 | 29.05 | 30.01 | 27.66 | 29.13 |
| 357 | 20359 | 33.92 | 30.78 | 33.06 | 29.96 |
| 358 | 20360 | 36.26 | 33.85 | 34.75 | 36.35 |
| 359 | 20361 | 30.29 | 28.67 | 29.42 | 27.69 |
| 360 | 20362 | 27.54 | 28.74 | 26.62 | 28.20 |
| 361 | 20363 | 33.50 | 28.76 | 32.02 | 28.16 |
| 362 | 20364 | 37.13 | 29.39 | 35.58 | 30.94 |
| 363 | 20365 | 33.13 | 29.15 | 32.56 | 29.65 |
| 364 | 20366 | 30.50 | 28.88 | 29.24 | 28.95 |
| 365 | 20367 | 30.04 | 29.05 | 29.95 | 28.66 |
| 366 | 20368 | 28.60 | 30.53 | 28.35 | 29.55 |
| 367 | 20369 | - | 29.95 | - | 29.14 |
| 368 | 20370 | 35.15 | 30.57 | 34.65 | 29.94 |
| 369 | 30001 | 37.78 | 31.36 | 37.88 | 31.53 |
| 370 | 30002 | - | 27.66 | - | 28.11 |
| 371 | 30003 | - | 33.28 | - | 35.58 |
| 372 | 30004 | 36.65 | 32.54 | 36.31 | 32.95 |
| 373 | 30005 | - | 27.75 | - | 27.49 |
| 374 | 30006 | - | 28.69 | - | 27.28 |
| 375 | 30007 | - | 29.77 | - | 28.94 |
| 376 | 30008 | - | 31.94 | - | 31.48 |
| 377 | 30009 | - | 27.38 | - | 27.40 |
| 378 | 30010 | - | 28.64 | - | 28.27 |
| 379 | 30011 | - | 33.49 | - | 33.84 |
| 380 | 30012 | - | 30.97 | - | 29.98 |
| 381 | 30013 | - | 27.80 | - | 27.35 |
| 382 | 30014 | - | 30.78 | 37.39 | 30.27 |
| 383 | 30015 | - | 32.11 | - | 33.00 |
| 384 | 30016 | - | 28.95 | - | 28.71 |
| 385 | 30017 | - | 29.58 | - | 29.60 |
| 386 | 30018 | - | 29.47 | - | 28.52 |
| 387 | 30019 | - | 30.58 | 38.23 | 29.67 |
| 388 | 30020 | - | 26.72 | - | 26.77 |
| 389 | 30021 | - | 34.99 | - | 33.58 |
| 390 | 30022 | 38.05 | 31.91 | 34.52 | 30.48 |
| 391 | 30023 | - | 24.88 | - | 24.68 |
| 392 | 30024 | - | 28.78 | - | 29.73 |
| 393 | 30025 | - | 29.65 | - | 30.45 |
| 394 | 30026 | - | 27.52 | - | 27.36 |
| 395 | 30027 | 33.46 | 27.47 | 34.23 | 27.36 |
| 396 | 30028 | - | 31.61 | - | 29.09 |
| 397 | 30029 | - | 28.34 | - | 25.72 |
| 398 | 30030 | - | 30.24 | - | 29.79 |
| 399 | 30031 | 37.14 | 31.60 | 36.47 | 32.45 |
| 400 | 30032 | - | 32.29 | - | 31.25 |
| 401 | 30033 | 34.10 | 30.19 | 26.82 | 28.68 |
| 402 | 30034 | - | 31.84 | - | 32.81 |
| 403 | 30035 | - | 31.45 | - | 31.44 |
| 404 | 30036 | - | 31.70 | - | 31.02 |
| 405 | 30037 | 29.04 | 32.92 | 28.16 | 34.63 |
| 406 | 30038 | - | 32.64 | - | 32.66 |
| 407 | 30039 | - | 29.20 | - | 27.93 |
| 408 | 30040 | - | 31.03 | - | 31.09 |
| 409 | 30041 | 31.09 | 31.64 | 29.96 | 31.52 |
| 410 | 30042 | - | 33.33 | 38.23 | 33.37 |
| 411 | 30043 | - | 31.48 | - | 31.60 |
| 412 | 30044 | - | 28.31 | - | 28.38 |
| 413 | 30045 | 25.65 | 30.86 | 24.18 | 31.66 |
| 414 | 30046 | 34.24 | 31.58 | 32.87 | 33.64 |
| 415 | 30047 | 33.90 | 29.95 | 32.19 | 31.20 |
| 416 | 30048 | 30.87 | 32.87 | 29.10 | 33.05 |
| 417 | 30049 | - | 29.10 | - | 29.99 |
| 418 | 30050 | - | 26.76 | - | 26.06 |
| 419 | 30051 | 32.67 | 33.26 | 31.74 | 34.08 |
| 420 | 30052 | - | 33.60 | - | 32.71 |
| 421 | 30053 | 27.63 | 30.80 | 26.64 | 31.65 |
| 422 | 30054 | 31.60 | 30.76 | 30.23 | 30.21 |
| 423 | 30055 | 33.30 | 33.70 | 31.69 | 32.03 |
| 424 | 30056 | 33.61 | 31.27 | 32.57 | 31.24 |
| 425 | 30057 | 25.13 | 30.08 | 24.79 | 30.90 |
| 426 | 30058 | - | 30.96 | - | 30.02 |
| 427 | 30059 | - | 33.51 | - | 32.08 |
| 428 | 30060 | 28.19 | 33.12 | 27.17 | 32.54 |
| 429 | 30061 | 30.77 | 30.01 | 29.93 | 30.06 |
| 430 | 30062 | - | 29.63 | - | 30.05 |
| 431 | 30063 | 26.01 | 29.01 | 25.39 | 29.23 |
| 432 | 30064 | 25.12 | 30.71 | 23.46 | 30.55 |
| 433 | 30065 | - | 29.38 | - | 30.27 |
| 434 | 30066 | - | 30.93 | - | 31.84 |
| 435 | 30067 | - | 29.18 | - | 28.34 |
| 436 | 30068 | - | 32.16 | - | 31.95 |
| 437 | 30069 | - | 29.15 | - | 28.69 |
| 438 | 30070 | - | 30.16 | - | 28.90 |
| 439 | 30071 | 26.71 | 28.61 | 25.51 | 30.43 |
| 440 | 30072 | 25.66 | 28.94 | 24.31 | 30.15 |
| 441 | 30073 | 33.50 | 33.30 | 32.32 | 30.61 |
| 442 | 30074 | 28.55 | 31.27 | 27.36 | 29.34 |
| 443 | 30075 | - | 33.12 | - | 33.71 |
| 444 | 30076 | 25.11 | 32.90 | 23.07 | 31.55 |
| 445 | 30077 | 38.47 | 33.15 | - | 32.05 |
| 446 | 30078 | 30.67 | 32.80 | 28.61 | 32.40 |
| 447 | 30079 | 28.99 | 31.40 | 27.24 | 30.91 |
| 448 | 30080 | 28.77 | 28.60 | 27.03 | 28.67 |
| 449 | 30081 | 29.15 | 29.80 | 28.18 | 30.17 |
| 450 | 30082 | 37.29 | 29.60 | 37.17 | 29.27 |
| 451 | 30083 | - | 30.37 | - | 30.15 |
| 452 | 30084 | - | 34.31 | - | 34.48 |
| 453 | 30085 | 38.21 | 30.76 | 36.49 | 32.67 |
| 454 | 30086 | - | 32.61 | - | 32.03 |
| 455 | 30087 | - | 29.60 | - | 27.31 |
| 456 | 30088 | - | 29.67 | - | 28.10 |
| 457 | 30089 | - | 32.53 | - | 33.55 |
| 458 | 30090 | - | 31.57 | - | 31.79 |
| 459 | 30091 | 31.25 | 32.78 | 30.71 | 33.17 |
| 460 | 30092 | 27.41 | 32.01 | 26.39 | 32.91 |
| 461 | 30093 | 24.05 | 33.41 | 23.21 | 32.74 |
| 462 | 30094 | - | 30.74 | - | 29.33 |
| 463 | 30095 | 30.33 | 27.55 | 29.41 | 26.15 |
| 464 | 30096 | 31.18 | 33.72 | 30.05 | 35.44 |
| 465 | 30097 | - | 34.50 | - | 35.45 |
| 466 | 30098 | - | 29.89 | - | 29.24 |
| 467 | 30099 | - | 31.42 | - | 31.97 |
| 468 | 30100 | 35.80 | 33.50 | 34.46 | 33.09 |
| 469 | 30101 | 35.01 | 31.96 | 34.01 | 31.71 |
| 470 | 30102 | - | 29.74 | - | 29.12 |
| 471 | 30103 | 35.93 | 32.70 | 36.28 | 30.13 |
| 472 | 30104 | - | 31.36 | - | 31.23 |
| 473 | 30105 | 32.11 | 29.22 | 30.87 | 29.67 |
| 474 | 30106 | 31.65 | 28.34 | 30.31 | 28.64 |
| 475 | 30107 | 35.15 | 31.72 | 33.33 | 31.65 |
| 476 | 30108 | 37.95 | 30.58 | 36.04 | 31.47 |
| 477 | 30109 | 27.76 | 30.68 | 26.73 | 29.68 |
| 478 | 30110 | 35.58 | 32.04 | 35.55 | 33.04 |
| 479 | 30111 | 27.59 | 30.88 | 26.05 | 30.43 |
| 480 | 30112 | - | 32.29 | - | 32.42 |
| 481 | 30113 | - | 32.30 | - | 31.74 |
| 482 | 30114 | 31.25 | 26.67 | 30.30 | 25.92 |
| 483 | 30115 | 35.46 | 30.88 | 34.93 | 31.58 |
| 484 | 30116 | - | 32.52 | - | 33.23 |
| 485 | 30117 | - | 33.27 | - | 32.16 |
| 486 | 30118 | 31.49 | 32.30 | 29.35 | 32.63 |
| 487 | 30119 | - | 32.87 | - | 33.00 |
| 488 | 30120 | 36.06 | 30.75 | 34.82 | 30.80 |
| 489 | 30121 | 27.89 | 31.18 | 26.46 | 31.34 |
| 490 | 30122 | 35.44 | 32.69 | 34.03 | 33.07 |
| 491 | 30123 | 35.12 | 31.22 | 33.78 | 31.13 |
| 492 | 30124 | 28.46 | 32.57 | 27.19 | 31.28 |
| 493 | 30125 | 38.95 | 31.94 | - | 32.39 |
| 494 | 30126 | - | 33.37 | - | 33.64 |
| 495 | 30127 | 30.35 | 31.05 | 28.73 | 30.99 |
| 496 | 30128 | 34.96 | 29.71 | 34.27 | 29.02 |
| 497 | 30129 | - | 31.76 | - | 30.10 |
| 498 | 30130 | 30.73 | 30.02 | 29.46 | 29.09 |
| 499 | 30131 | 32.25 | 32.73 | 31.18 | 33.13 |
| 500 | 30132 | - | 28.11 | - | 25.78 |
| 501 | 30133 | 23.69 | 30.65 | 22.29 | 30.09 |
| 502 | 30134 | - | 29.52 | - | 27.79 |
| 503 | 30135 | - | 31.92 | 36.44 | 30.48 |
| 504 | 30136 | 31.10 | 31.35 | 29.28 | 30.28 |
| 505 | 30137 | - | 31.78 | - | 32.34 |
| 506 | 30138 | - | 33.82 | - | 33.43 |
| 507 | 30139 | - | 31.36 | - | 31.26 |
| 508 | 30140 | - | 32.48 | - | 31.47 |
| 509 | 30141 | 32.29 | 32.31 | 30.33 | 30.02 |
| 510 | 30142 | 29.37 | 29.76 | 28.09 | 28.43 |
| 511 | 30143 | 32.08 | 30.14 | 30.29 | 29.96 |
| 512 | 30144 | 35.90 | 34.22 | 33.44 | 33.75 |
| 513 | 30145 | - | 32.90 | - | 34.23 |
| 514 | 30146 | - | 32.70 | - | 33.55 |
| 515 | 30147 | 37.02 | 34.32 | 37.21 | 34.72 |
| 516 | 30148 | - | 33.15 | - | 33.22 |
| 517 | 30149 | 38.03 | 32.61 | 37.02 | 30.63 |
| 518 | 30150 | 35.99 | 33.74 | 33.74 | 33.52 |
| 519 | 30151 | 35.05 | 34.09 | 33.22 | 32.95 |
| 520 | 30152 | - | 33.02 | - | 34.92 |
| 521 | 30153 | - | 29.42 | - | 28.07 |
| 522 | 30154 | - | 31.17 | - | 31.34 |
| 523 | 30155 | 27.96 | 32.18 | 27.04 | 31.85 |
| 524 | 30156 | - | 34.40 | - | 34.43 |
| 525 | 30157 | - | 29.70 | - | 31.38 |

Abbreviations: No., number; FME, five-minute nucleic acid extraction; Ct, cycle threshold; IAV, influenza A virus; RNase P, ribonuclease P; -, negative.
