# Supplementary figures and images for: Development and validation of a rapid five-minute nucleic acid extraction method for respiratory viruses
Source: Virol J. 2024 Aug 18;21:189. doi: 10.1186/s12985-024-02381-3 (PMC11331601; doi:10.1186/s12985-024-02381-3)

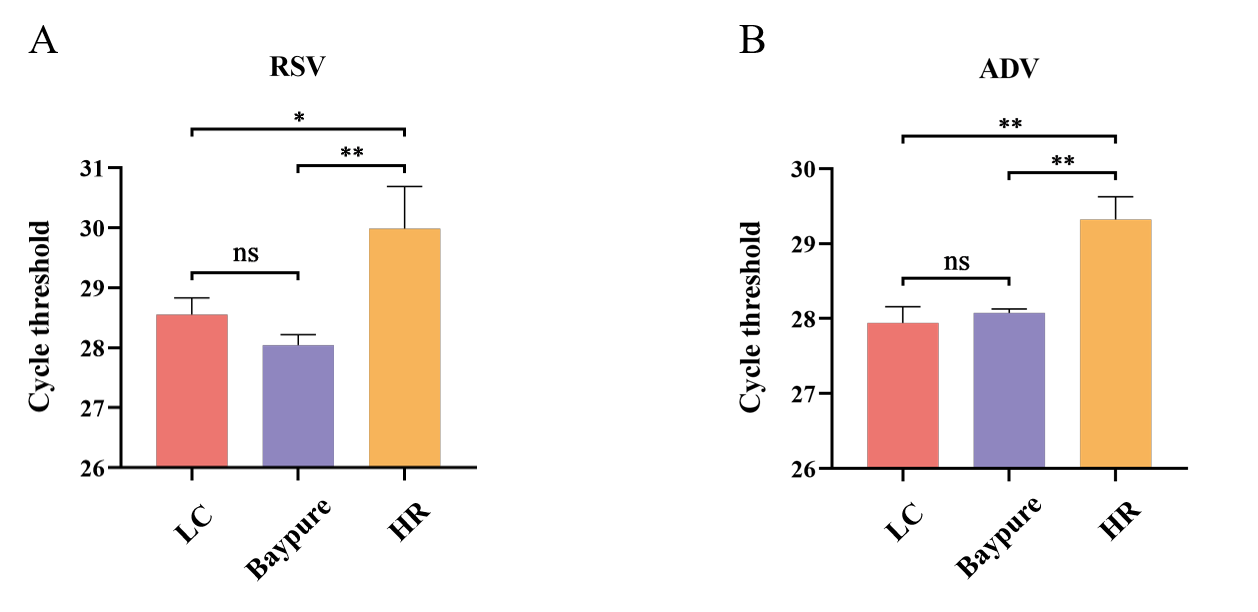

Supplement: Supplementary file 2 — Additional file 2: Figure S1. The LC exhibits an extraction efficiency similar to the magnetic bead method. A and B RSV and ADV nucleic acid were extracted using LC, Baypure, and HR magnetic bead extraction kits, and qRT-PCR Ct values were used to assess the efficiency of each method for extracting nucleic acid. A: Effect of RSV nucleic acid extraction. B: Effect of ADV nucleic acid extraction. Data are presented as means± SD for three independent biological replicates. Statistical significance was calculated using t tests; ns P > 0.05, *P < 0.05, **P < 0.01. [file 12985_2024_2381_MOESM2_ESM.tif]

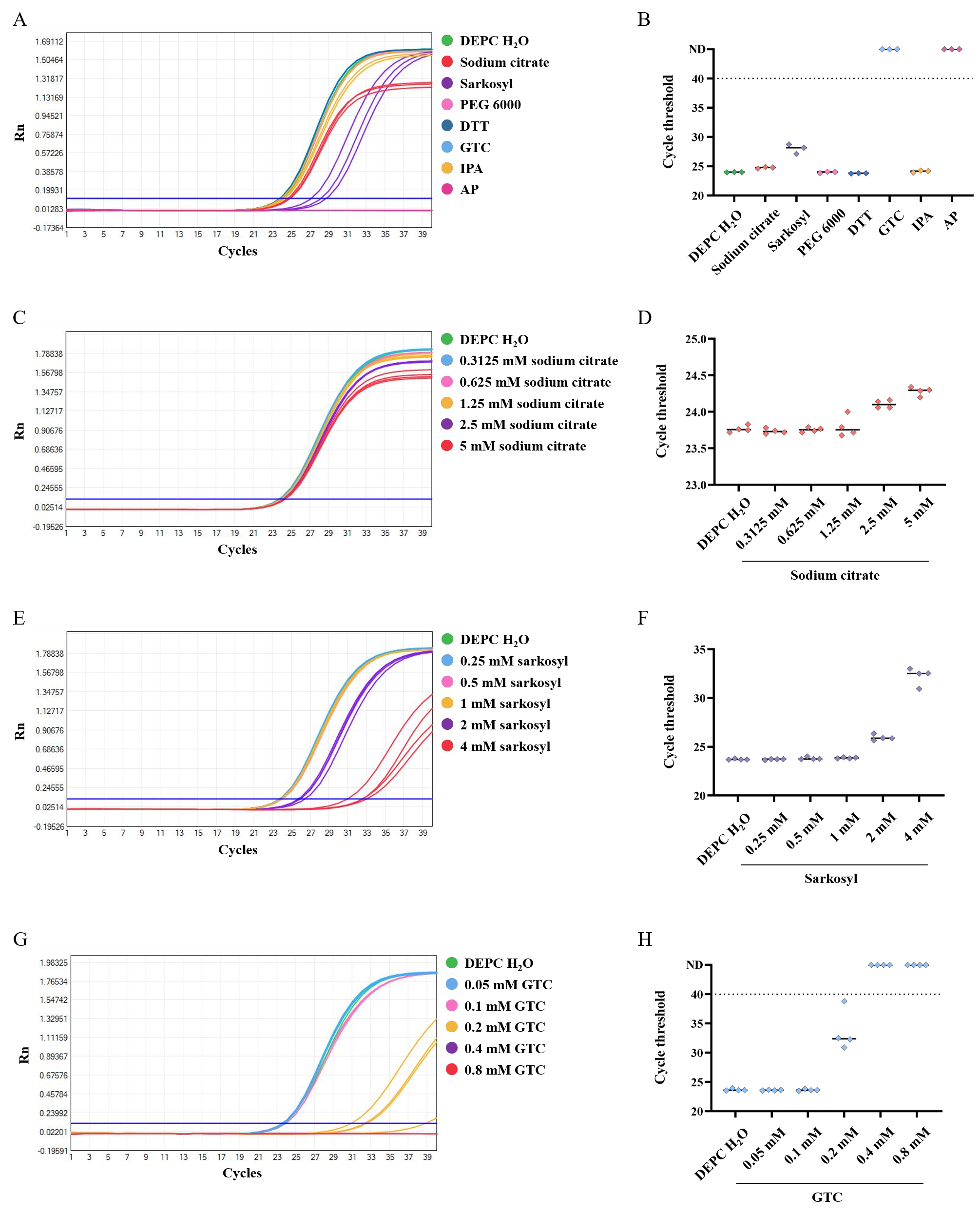

Supplement: Supplementary file 3 — Additional file 3: Figure S2. Effect of residual AP lysis solution on PCR amplification. RSV nucleic acid was extracted at a high concentration by the FME. A and B In the RSV reaction system, 4 μL of RSV nucleic acid was added, along with an additional 1 μL each of DEPC H2O, 25 mM sodium citrate, 20 mM sarkosyl, 2.5% PEG 6000, 1 M DTT, and 4 M GTC, IPA, or AP lysis solution. qRT-PCR Ct values were used to assess the effect of the residual AP lysis solution components on amplification. C and D 4 μL of RSV nucleic acid was added, followed by an additional 1 μL of sodium citrate diluted in a twofold gradient. Ct values were used to assess the effect of residual sodium citrate on PCR amplification. E and F 4 μL of RSV nucleic acid was added, followed by an additional 1 μL of sarkosyl diluted in a twofold gradient. Ct values were used to assess the effect of residual sarkosyl on PCR amplification. G and H 4 μL of RSV nucleic acid was added, followed by an additional 1 μL of GTC in a twofold gradient. Ct values were used to assess the effect of GTC on PCR amplification. Solid lines indicate the median, and dashed lines indicate the detection limit. Data are presented as means ± SD for three or four independent biological replicates. [file 12985_2024_2381_MOESM3_ESM.tif]

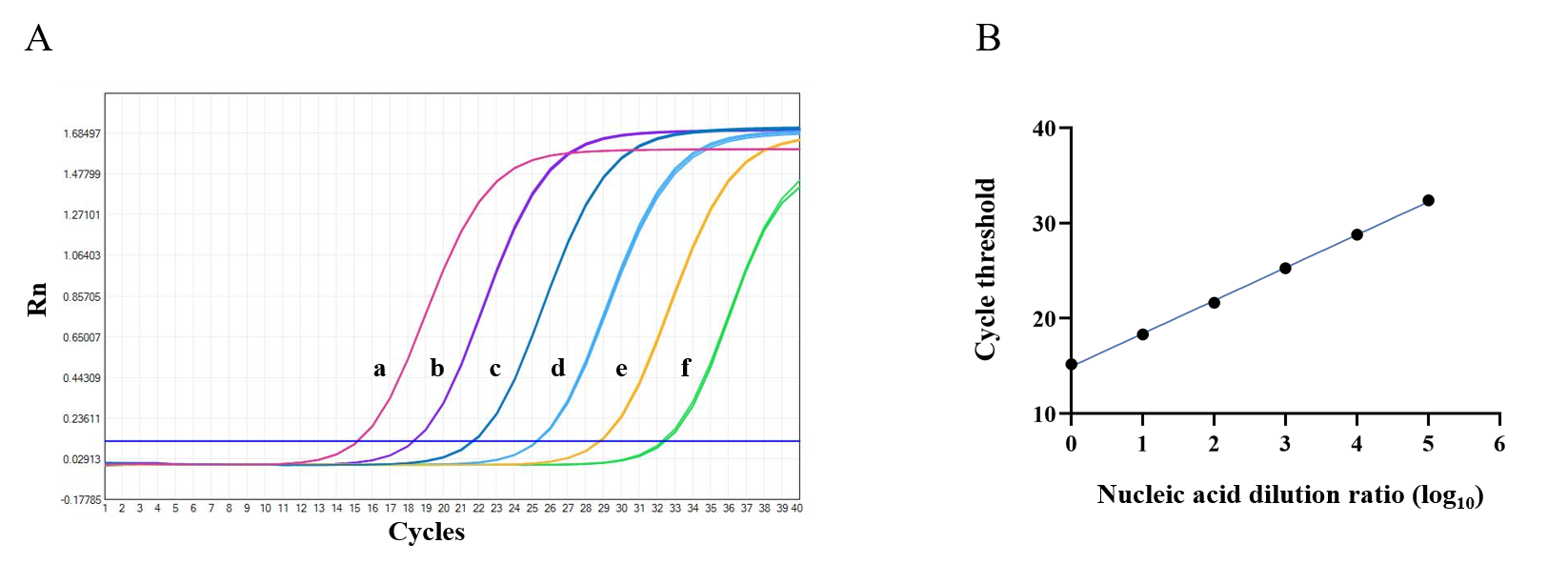

Supplement: Supplementary file 4 — Additional file 4: Figure S3. The Ct values of the eluted nucleic acid were determined following a 10-fold gradient dilution. High concentrations of RSV were extracted by the FME, and the nucleic acid in the elution was sequentially diluted using a 10-fold gradient with DEPC H2O. The Ct values were determined by qRT-PCR for each dilution gradient, with each measurement repeated three times. A Amplification curve. B Linear regression curve, R2= 0.9993. [file 12985_2024_2381_MOESM4_ESM.tif]

**Fig. 3B** DNA integrity analysis of the FME system


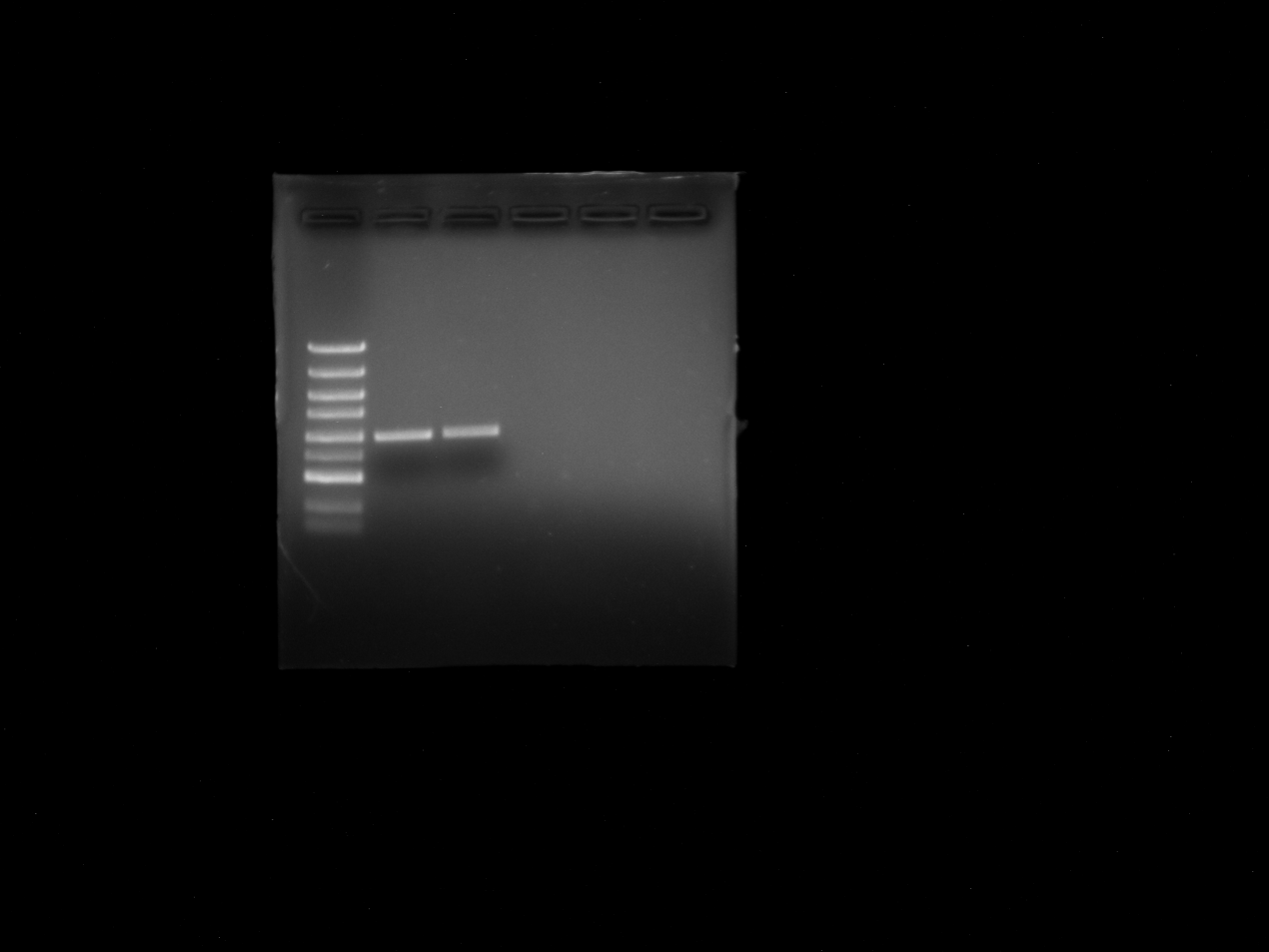

Supplement: Supplementary file 6 — Additional file 6. [file 12985_2024_2381_MOESM6_ESM.docx]
